# Assessing real-world gait with digital technology? Validation, insights and recommendations from the Mobilise-D consortium

**DOI:** 10.1186/s12984-023-01198-5

**Published:** 2023-06-14

**Authors:** M. Encarna Micó-Amigo, Tecla Bonci, Anisoara Paraschiv-Ionescu, Martin Ullrich, Cameron Kirk, Abolfazl Soltani, Arne Küderle, Eran Gazit, Francesca Salis, Lisa Alcock, Kamiar Aminian, Clemens Becker, Stefano Bertuletti, Philip Brown, Ellen Buckley, Alma Cantu, Anne-Elie Carsin, Marco Caruso, Brian Caulfield, Andrea Cereatti, Lorenzo Chiari, Ilaria D’Ascanio, Bjoern Eskofier, Sara Fernstad, Marcel Froehlich, Judith Garcia-Aymerich, Clint Hansen, Jeffrey M. Hausdorff, Hugo Hiden, Emily Hume, Alison Keogh, Felix Kluge, Sarah Koch, Walter Maetzler, Dimitrios Megaritis, Arne Mueller, Martijn Niessen, Luca Palmerini, Lars Schwickert, Kirsty Scott, Basil Sharrack, Henrik Sillén, David Singleton, Beatrix Vereijken, Ioannis Vogiatzis, Alison J. Yarnall, Lynn Rochester, Claudia Mazzà, Silvia Del Din, for the Mobilise-D consortium

**Affiliations:** 1 Translational and Clinical Research Institute, Faculty of Medical Sciences, Newcastle University, Newcastle upon Tyne, UK; 2 Department of Mechanical Engineering and Insigneo Institute for in Silico Medicine, The University of Sheffield, Sheffield, UK; 3 Laboratory of Movement Analysis and Measurement, Ecole Polytechnique Federale de Lausanne, Lausanne, Switzerland; 4 Machine Learning and Data Analytics Lab, Department of Artificial Intelligence in Biomedical Engineering, Friedrich-Alexander-Universität Erlangen-Nürnberg, Erlangen, Germany; 5 Center for the Study of Movement, Cognition and Mobility, Neurological Institute, Tel Aviv Sourasky Medical Center, Tel Aviv, Israel; 6 Department of Biomedical Sciences, University of Sassari, Sassari, Italy; 7 National Institute for Health and Care Research (NIHR) Newcastle Biomedical Research Centre (BRC), Newcastle University and The Newcastle upon Tyne Hospitals NHS Foundation Trust, Newcastle upon Tyne, UK; 8 Robert Bosch Gesellschaft für Medizinische Forschung, Stuttgart, Germany; 9 Department of Electronics and Telecommunications, Politecnico di Torino, Turin, Italy; 10 The Newcastle Upon Tyne Hospitals NHS Foundation Trust, Newcastle upon Tyne, UK; 11 School of Computing, Newcastle University, Newcastle upon Tyne, UK; 12 Barcelona Institute for Global Health (ISGlobal), Barcelona, Spain; 13 Universitat Pompeu Fabra, Barcelona, Catalonia Spain; 14 CIBER Epidemiología y Salud Pública (CIBERESP), Madrid, Spain; 15 Insight Centre for Data Analytics, University College Dublin, Dublin, Ireland; 16 School of Public Health, Physiotherapy and Sports Science, University College Dublin, Dublin, Ireland; 17 Department of Electrical, Electronic and Information Engineering «Guglielmo Marconi», University of Bologna, Bologna, Italy; 18 Health Sciences and Technologies—Interdepartmental Center for Industrial Research (CIRI-SDV), University of Bologna, Bologna, Italy; 19 Grünenthal GmbH, Aachen, Germany; 20 Department of Neurology, University Medical Center Schleswig-Holstein Campus Kiel, Kiel, Germany; 21 Sagol School of Neuroscience and Department of Physical Therapy, Sackler Faculty of Medicine, Tel Aviv University, Tel Aviv, Israel; 22 Rush Alzheimer’s Disease Center and Department of Orthopaedic Surgery, Rush University Medical Center, Chicago, IL USA; 23 Department of Sport, Exercise and Rehabilitation, Northumbria University Newcastle, Newcastle upon Tyne, UK; 24 Novartis Institutes of Biomedical Research, Novartis Pharma AG, Basel, Switzerland; 25 McRoberts BV, The Hague, Netherlands; 26 Department of Neuroscience and Sheffield NIHR Translational Neuroscience BRC, Sheffield Teaching Hospitals NHS Foundation Trust, Sheffield, UK; 27 Digital Health R&D, AstraZeneca, Stockholm, Sweden; 28 Department of Neuromedicine and Movement Science, Norwegian University of Science and Technology, Trondheim, Norway

**Keywords:** Real-world gait, Algorithms, DMOs, Validation, Wearable sensor, Walking, Cadence, SL, Digital health, Accelerometer

## Abstract

**Background:**

Although digital mobility outcomes (DMOs) can be readily calculated from real-world data collected with wearable devices and ad-hoc algorithms, technical validation is still required. The aim of this paper is to comparatively assess and validate DMOs estimated using real-world gait data from six different cohorts, focusing on gait sequence detection, foot initial contact detection (ICD), cadence (CAD) and stride length (SL) estimates.

**Methods:**

Twenty healthy older adults, 20 people with Parkinson’s disease, 20 with multiple sclerosis, 19 with proximal femoral fracture, 17 with chronic obstructive pulmonary disease and 12 with congestive heart failure were monitored for 2.5 h in the real-world, using a single wearable device worn on the lower back. A reference system combining inertial modules with distance sensors and pressure insoles was used for comparison of DMOs from the single wearable device. We assessed and validated three algorithms for gait sequence detection, four for ICD, three for CAD and four for SL by concurrently comparing their performances (e.g., accuracy, specificity, sensitivity, absolute and relative errors). Additionally, the effects of walking bout (WB) speed and duration on algorithm performance were investigated.

**Results:**

We identified two cohort-specific top performing algorithms for gait sequence detection and CAD, and a single best for ICD and SL. Best gait sequence detection algorithms showed good performances (sensitivity > 0.73, positive predictive values > 0.75, specificity > 0.95, accuracy > 0.94). ICD and CAD algorithms presented excellent results, with sensitivity > 0.79, positive predictive values > 0.89 and relative errors < 11% for ICD and < 8.5% for CAD. The best identified SL algorithm showed lower performances than other DMOs (absolute error < 0.21 m). Lower performances across all DMOs were found for the cohort with most severe gait impairments (proximal femoral fracture).

Algorithms’ performances were lower for short walking bouts; slower gait speeds (< 0.5 m/s) resulted in reduced performance of the CAD and SL algorithms.

**Conclusions:**

Overall, the identified algorithms enabled a robust estimation of key DMOs. Our findings showed that the choice of algorithm for estimation of gait sequence detection and CAD should be cohort-specific (e.g., slow walkers and with gait impairments). Short walking bout length and slow walking speed worsened algorithms’ performances.

*Trial registration* ISRCTN – 12246987.

**Supplementary Information:**

The online version contains supplementary material available at 10.1186/s12984-023-01198-5.

## Introduction

The adverse consequences of physical mobility loss and the importance of preserving mobility to ensure healthy ageing are undeniable [1, 2]. For this reason, a variety of behavioural, nutritional, and pharmacological interventions aim to improve mobility in general, and more specifically target the preservation of an individual’s ability to walk independently and safely both within and outside their homes [3–6]. Evaluating the effectiveness of interventions by quantifying an improved gait pattern, however, remains a challenge when relying on traditional tools such as patient-reported outcomes or supervised gait tests in clinic or lab, as these typically lack ecological validity [7].

Therefore, there is a need for the development of accurate, reliable, and sensitive tools for the quantification of gait and mobility in real-life [8, 9]. Digital health technology, including body-worn or wearable devices, offers a way forward by providing digital outcomes to remotely measure and monitor gait [10, 11], a fundamental component of mobility [12, 13]. Nonetheless, due to several persisting challenges in this field, current tools and techniques are still in their infancy. These challenges need to be addressed before digital mobility outcomes can be confidently adopted in clinical trials and as part of standard healthcare, including a variety of technical, clinical, and regulatory aspects [9, 14].

Exciting technical advances in algorithms and data processing techniques have led to the deployment of a plethora of algorithms to extract digital mobility outcomes from gait data recorded using inertial measurement units embedded within wearable devices [15–17]. Even so, significant ongoing challenges exist, in particular establishing the technical validity of these algorithms. A thorough validation process must account for complex factors that simultaneously arise from multiple sources influencing digital mobility outcome measures, including disease characteristics, patient specific habits, and the context in which walking is recorded (i.e. indoors, outdoors, public vs. private domain) [18–20]. All these factors concur to potentially limit the generalizability of validation data recorded during traditional gait protocols such as those administered within a controlled clinical or laboratory setting in which participants are asked to walk along a straight path or just a few daily life activities are simulated [21, 22]. Only recently, ad-hoc wearable devices have been developed, which finally allow moving the validation to more complex and realistic real-life scenarios [19, 23]. However, published validation studies generally only target a subset of specific digital mobility outcomes as calculated from one or a reduced number of algorithms and/or include only a few cohorts, hence providing partial information about generalizability of the results [22, 24].

The aim of this paper is to identify, compare and rank the most promising algorithms that quantitatively characterize gait with digital mobility outcomes from continuous real-life monitoring in a diverse group of patients who present with different mobility challenges. Here we focus on detection of gait sequences (i.e., identified walking bouts), individual steps, and estimation of cadence and stride length from a single wearable device positioned on the lower back, an ergonomically easy-to-use position near the centre of mass, which is well accepted by participants [25, 26]. To establish generalizability, we independently compare algorithms in six cohorts: healthy older adults, Parkinson’s disease, multiple sclerosis, proximal femoral fracture, chronic obstructive pulmonary disease and congestive heart failure. Specifically, we aim to:

- Identify, compare and rank the best performing (i.e., most accurate and reliable) algorithms for each cohort;
- Describe the performance of the identified best algorithms;
- Analyse the influence of walking speed and walking bout duration on the algorithm performance;
- Provide recommendations to implement and select algorithms for real-world gait analysis tailored to different patient cohorts.

## Methods

### Participants

A convenience sample of 108 participants were recruited to represent five disease cohorts (chronic obstructive pulmonary disease, Parkinson’s disease, multiple sclerosis, proximal femoral fracture, and congestive heart failure), as well as healthy older adults, encompassing a broad range of mobility levels. Participants were recruited in five sites: The Newcastle upon Tyne Hospitals NHS Foundation Trust, UK and Sheffield Teaching Hospitals NHS Foundation Trust, UK (ethics approval granted by London – Bloomsbury Research Ethics committee, 19/LO/1507); Tel Aviv Sourasky Medical Center, Israel (ethics approval granted by the Helsinki Committee, Tel Aviv Sourasky Medical Center, Tel Aviv, Israel, 0551-19TLV), Robert Bosch Foundation for Medical Research, Germany (ethics approval granted by the ethical committee of the medical faculty of The University of Tübingen, 647/2019BO2), University of Kiel, Germany (ethics approval granted by the ethical committee of the medical faculty of Kiel University, D438/18). All participants gave written informed consent to take part in the study. Inclusion and exclusion criteria and details about the technical validation study experimental protocol are described in [19].

### Experimental protocol

Participants were monitored for 2.5 h as they went about their usual activities in their habitual environment (home/work/community/outdoor). To ensure diversity of walking activity, participants were also asked to perform some specific tasks: outdoor walking; walking up and down a slope and stairs; and moving from one room to another. Participants wore a single McRoberts Dynaport MM+ wearable device (sampling frequency 100 Hz, triaxial acceleration range: ± 8 g/resolution: 1 mg, triaxial gyroscope range: ± 2000 degrees per second (dps)/resolution: 70 mdps), secured to the lower back with an elasticated belt and Velcro fastening. A reference system was used to establish the accuracy of algorithms and was comprised of a multicomponent system of INertial modules, DIstance Sensors and Pressure insoles (INDIP) [19, 23, 27]. The INDIP system and the associated algorithms to estimate digital mobility outcomes have been validated in previous studies in healthy and pathological cohorts (e.g., hemiparetic, Parkinson’s disease, Huntington’s disease and mild cognitive impairment) and in this study participants [23, 28–32]. The INDIP and the single wearable device on the lower back were synchronized using timestamps referred to a common clock [19].

### Pre-selection of algorithms for further validation and ranking

In this paper we focused on key metrics of real-world walking that form the basis from which a variety of digital mobility outcomes, including walking speed, can then be quantified. These are: gait sequence detection, foot initial contact detection, cadence and stride length estimation. For each metric, we identified published algorithms from laboratory-based or semi-structured protocols [8, 33]. This yielded 14 for gait sequence detection, 21 for initial contact detection, 23 for cadence and 18 for stride length estimation. For each digital mobility outcome, a shortlist of up to four most promising algorithms was selected based on initial testing in pre-existing data from older adults and pathological cohorts, including Parkinson’s disease [28, 34–36], multiple sclerosis [37, 38], stroke & chorea [28, 39]. Algorithms’ selection was based on the ranking methodology proposed in Bonci et al. [24]. The final subset of optimized algorithms (including detailed descriptions of implementation) are summarized in Table 1 and briefly outlined below.

**Table 1:** Description of algorithms for each metric: gait sequence detection (GSD), initial contact event detection (ICD), cadence estimation (CAD) and stride length estimation (SL)

Metric	Name	Description	Input	Output	Language	References
Gait sequence detection	GSD_A_	Based on a frequency-based approach, this algorithm is implemented on the vertical and anterior–posterior acceleration signals. First, these are band pass filtered to keep frequencies between 0.5 and 3 Hz. Next, a convolution of a 2 Hz sinewave (representing a template for a gait cycle) is performed, from which local maxima will be detected to define the regions of gait	*acc_v*: vertical acceleration *acc_ap*: anterior–posterior acceleration *WinS* = *3 s;* window size for convolution *OL* = *1.5 s;* overlap of windows *Activity_thresh* = *0.01;* Motion threshold *Fs:* sampling frequency	*Start:* beginning of N gait sequences [s] relative to the start of a recording or a test/trial. Format: 1 × N vector *End:* termination of N gait sequences [s] relative to the start of a recording or a test/trial. Format: 1 × N vector	Matlab®	Iluz, Gazit [40]
	GSD_B_	This algorithm, based on a time domain-approach, detects the gait periods based on identified steps. First, the norm of triaxial acceleration signal is low-pass filtered (FIR, fc=3.2Hz), then a peak detection procedure using a threshold of 0.1 [g] is applied to identify steps. Consecutive steps, detected using an adaptive step duration threshold are associated to gait sequences.	*acc_norm*: norm of the 3D-accelerometer signal *Fs:* sampling frequency *th*: peak detection threshold: 0.1 (g)	*Start:* beginning of N gait sequences [s] relative to the start of a recording or a test/trial. Format: 1 x N vector *End:* termination of N gait sequences [s] relative to the start of a recording or a test/trial. Format: 1 x N vector	Matlab®	Paraschiv-Ionescu, Newman [41]
	GSD_c_	This algorithm utilizes the same approach as GSDB the only difference being a different threshold for peak detection of 0.15 [g]	*acc_norm*: norm of the 3D-accelerometer signal *Fs:* sampling frequency *th:* peak detection threshold: 0.15 (g)	*Start:* beginning of N gait sequences [s] relative to the start of a recording or a test/trial. Format: 1 × N vector *End:* termination of N gait sequences [s] relative to the start of a recording or a test/trial. Format: 1 × N vector	Matlab®	Paraschiv-Ionescu, Newman [41]
Initial Contact Detection	ICD_A_	Algorithm implemented on a pre-processed *vertical* acceleration signal. This is first detrended and then low-pass filtered (FIR, f_c_ = 3.2 Hz). The resulting signal is numerically integrated (*cumtrapz*) and differentiated using a Gaussian continuous wavelet transformation (CWT, scale 9, gauss2). The initial contact events are identified as the positive peaks between zero-crossings	*acc_v:* the acceleration in the vertical axis *Fs:* sampling frequency *GS:* array of gait sequences	*ICs*: detected N initial contacts [s] relative to the beginning of the recording or the test/trial. Format: 1 × N vector *GaitSequenceRefined_Start:* refinement of the beginning of the gait sequences provided from the *Gait sequence detection* step; it starts one second before the first detected IC *GaitSequenceRefined_End*: refinement of the termination of the gait sequences provided from the *Gait sequence detection* step; it finishes one second after the last detected IC	Matlab®	McCamley, Donati [42] Paraschiv-Ionescu, Newman [41] Paraschiv-Ionescu, Soltani [16]
	ICD_B_	Algorithm implemented on a pre-processed *anterior–posterior* acceleration signal. This is first detrended and then low-pass filtered at 10 Hz with a second-order Butterworth filter. The resulting signal is numerically integrated (*cumtrapz*) and differentiated using an estimated wavelet scale [43] and a Gaussian first-order (gaus1) wavelet. The initial contact events are identified with a peak detection function (*findpeaks*)	*acc_ap*: the acceleration in the anterior–posterior axis *Fs:* sampling frequency *GS:* array of gait sequences	*ICs*: detected N initial contacts [s] relative to the beginning of the recording or the test/trial. Format: 1 × N vector *GaitSequenceRefined_Start:* refinement of the beginning of the gait sequences provided from the *Gait sequence detection* step; it starts one second before the first detected IC *GaitSequenceRefined_End*: refinement of the termination of the gait sequences provided from the *Gait sequence detection* step; it finishes one second after the last detected IC	Matlab®	McCamley, Donati [42] Abry [43] Pham, Elshehabi [44]
	ICD_C_	This algorithm is an optimized version of algorithm ICD_A_. In this case, the pre-processing of the acceleration signal is selected according to the degree of gait impairment, assessed with the symmetry index (ref) ∙ If estimated symmetry index > 0.5 the pre-processing of acceleration includes, in the following order: detrending and low-pass filtering (FIR, fc = 3.2 Hz), numerical integration (*cumtrapz*) and differentiation using Gaussian continuous wavelet transformation (CWT, scale 9, gauss2); ∙ If estimated symmetry index is in the range [0.25–0,5] the pre-processing includes an additional smoothing using moving average after CWT (*smoothdata*) ∙ If estimated symmetry index < 0.25, i.e., very impaired gait, the pre-processing includes *two* successive moving average after CWT, to efficiently smooth the signal	*acc_v:* the acceleration in the vertical axis *Fs:* sampling frequency *GS:* array of gait sequences	*ICs*: detected N initial contacts [s] relative to the beginning of the recording or the test/trial. Format: 1 × N vector *GaitSequenceRefined_Start:* refinement of the beginning of the gait sequences provided from the *Gait sequence detection* step; it starts one second before the first detected IC *GaitSequenceRefined_End*: refinement of the termination of the gait sequences provided from the *Gait sequence detection* step; it finishes one second after the last detected IC	Matlab®	McCamley, Donati [42] Paraschiv-Ionescu, Newman [41] Paraschiv-Ionescu, Soltani [16]
	ICD_D_	This algorithm is implemented on the norm of the 3D acceleration signals A sliding window summing technique is utilized to reduce the noise of the norm signal. Next, a differential technique on the acceleration is used to eliminate the effect of gravity, as a high pass filter. Finally, zero-crossings on the positive slope are detected, which determine the initial contact time events	*acc_norm*: norm of the 3d-accelerometer signal *Fs:* sampling frequency *GS:* array of gait sequences	*ICs*: detected N initial contacts [s] relative to the beginning of the recording or the test/trial. Format: 1 × N vector *GaitSequenceRefined_Start:* refinement of the beginning of the gait sequences provided from the *Gait sequence detection* step; it starts one second before the first detected IC *GaitSequenceRefined_End*: refinement of the termination of the gait sequences provided from the *Gait sequence detection* step; it finishes one second after the last detected IC	Matlab®	Shin and Park [45]
Cadence	CAD_A_	This algorithm is the same as ICD_A_. From the initial contact events estimated, the step periodicity is derived and the CAD is calculated	*acc_v:* the acceleration in the vertical axis *Fs:* sampling frequency *GS:* array of gait sequences	*output_CAD*: a vector structure containing: *output_CAD(N).start*: start of N-th GS [s] *output_CAD(N).stop*: stop of N-th GS [s] *output_CAD(N).cadSec*: Estimated CAD-per-sec [steps/min] of N-th GS *output_CAD(N).cadMean*: mean of CAD [steps/min] estimated for N-th GS *output_CAD(N).cadSTD*: std of CAD [steps/min] estimated for N-th GS *output_CAD(N).steps*: number of steps of N-th GS	Matlab®	McCamley, Donati [42] Paraschiv-Ionescu, Newman [41] Paraschiv-Ionescu, Soltani [16] Soltani, Aminian [17]
	CAD_B_	This algorithm first implements a pre-processing of the 3D acceleration norm, including low-pass filtering (FIR, fc = 3.2 Hz), detrending, smoothing using Savitsky–Golay filter (order = 7, frame length = 21) and Gaussian smoothing and finally a continuous wavelet transformation (CWT, scale 10, ‘gaus2’) to enhance the relevant steps-related features in acceleration signal. The pre-processed signal is then further processed using morphological filters, according to methods described in Lee, You [46]. Finally, the timing of steps, used to estimate step/stride frequency are detected as maxima between zero-crossings	*acc_norm*: norm of the 3D-accelerometer signal *Fs:* sampling frequency *GS:* array of gait sequences	*output_CAD*: a vector structure containing: *output_CAD(N).start*: start of N-th GS [s] *output_CAD(N).stop*: stop of N-th GS [s] *output_CAD(N).cadSec*: Estimated CAD-per-sec [steps/min] of N-th GS *output_CAD(N).cadMean*: mean of CAD [steps/min] estimated for N-th GS *output_CAD(N).cadSTD*: std of CAD [steps/min] estimated for N-th GS *output_CAD(N).steps*: number of steps of N-th GS	Matlab®	Lee, You [46] Paraschiv-Ionescu, Soltani [16] Soltani, Aminian [17]
	CAD_C_	This algorithm is the same as ICD_D_. From the initial contact events estimated, the step periodicity is derived and the CAD is calculated	*acc_norm*: norm of the 3D-accelerometer signal *Fs:* sampling frequency *GS:* array of gait sequences	*output_CAD*: a vector structure containing: *output_CAD(N).start*: start of N-th GS [s] *output_CAD(N).stop*: stop of N-th GS [s] *output_CAD(N).cadSec*: Estimated CAD-per-sec [steps/min] of N-th GS *output_CAD(N).cadMean*: mean of CAD [steps/min] estimated for N-th GS *output_CAD(N).cadSTD*: std of CAD [steps/min] estimated for N-th GS *output_CAD(N).steps*: Number of steps of N-th GS	Matlab®	Shin and Park [45] Soltani, Aminian [17]
Stride length	SL_A_	Biomechanical model which includes: – The implementation of the inverted pendulum model – Double integration of the acceleration in the vertical axis – Detrending and removal of integration drift: high pass filtering of vertical acceleration (f_c_ = 0.1 Hz) and integrated vertical acceleration (f_c_ = 1 Hz) – Correction factors based on the calibration factor “k”. Note that the tuning coefficients are optimized by training	*acc_v:* the acceleration in the vertical axis (orientation corrected) *Fs:* sampling frequency *GS:* array of gait sequences *ICs:* initial contacts [s] *Leg length* [cm] *Calibration factors “k”* (*k* = 4.739 for all cohorts)	*output_sl*: a vector structure containing: *output_sl(N).start*: start of N-th GS [s] *output_sl(N).stop*: stop of N-th GS [s] *output_sl(N).slSec*: Estimated SL-per-second [m] of N-th GS *output_sl(N).slMean*: mean of SL [m] estimated for N-th GS *output_sl(N).slSTD*: std of SL [m] estimated for N-th GS *output_sl(N).distanc*e: covered distance [m] of N-th GS *output_sl(N).warnMes*: shows if there is a warning for N-th GS (1 means yes)	Matlab®	Zijlstra and Hof [47] Zijlstra and Zijlstra [48] Soltani, Aminian [17]
	SL_B_ *SL_B_	This algorithm is the same as SL_A_. For all cohorts, the same factor “k” is used, whereas for the MS cohort, a special factor is implemented (*SL_B_)	*acc_v:* the acceleration in the vertical axis (orientation corrected) *Fs:* sampling frequency *GS:* array of gait sequences *ICs:* initial contacts [s] *Leg length* [cm] *Calibration factors* “*k*” (*k* = 4.99 for all cohorts except MS, for which *k* = 4.587)	*output_sl*: a vector structure containing: *output_sl(N).start*: start of N-th GS [s] *output_sl(N).stop*: stop of N-th GS [s] *output_sl(N).slSec*: Estimated SL-per-second [m] of N-th GS *output_sl(N).slMean*: mean of SL [m] estimated for N-th GS *output_sl(N).slSTD*: std of SL [m] estimated for N-th GS *output_sl(N).distanc*e: covered distance [m] of N-th GS *output_sl(N).warnMes*: shows if there is a warning for N-th GS (1 means yes)	Matlab®	Zijlstra and Hof [47] Zijlstra and Zijlstra [48] Soltani, Aminian [17]
	SL_C_	Based on the intensity of the vertical acceleration signal, this algorithm estimates the step length according to the following formulas, which represent the relation between the measured vertical acceleration, as a mean absolute value during a step cycle, and the samples within the step (ic = initial contact time event, k = calibration factor 1, p = calibration factor 2). Note that in this section, variables k and p are the tuning coefficients, optimized by machine-learning training ${a}_{v Mean \left(m\right)=mean \left|{a}_{v} \left(t\right)\right|}$ $ic \left(m\right)\le t \le ic (m+1)$ $SL=$ $t x \sqrt[3]{{a}_{v Mean (m)}}+p$	*acc_v:* the acceleration in the vertical axis (orientation corrected) *Fs:* sampling frequency *GS:* array of gait sequences *ICs:* initial contacts [s] *Leg length* [cm] *Calibration factors* “*t*” *and* “*p*” (*t* = 0.93, p = foot length)	*output_sl*: a vector structure containing: *output_sl(N).start*: start of N-th GS [s] *output_sl(N).stop*: stop of N-th GS [s] *output_sl(N).slSec*: Estimated SL-per-second [m] of N-th GS *output_sl(N).slMean*: mean of SL [m] estimated for N-th GS *output_sl(N).slSTD*: std of SL [m] estimated for N-th GS *output_sl(N).distanc*e: covered distance [m] of N-th GS *output_sl(N).warnMes*: shows if there is a warning for N-th GS (1 means yes)	Matlab®	Kim, Jang [49] Zhao, Zhang [50] Soltani, Aminian [17]
	SL_D_	This algorithm is based on the double integration of the anterior–posterior acceleration signal It integrates the signal and corrects the drift based on the combination of two methods: – The inverted pendulum model, as for SL_A_ – The geometrical acceleration-intensity-based model, which assumes the following: $SL=$ $t x \sqrt[4]{{a}_{v Max-Min (m)}}+p$ Note that in this section, variables k, t and p are the tuning coefficients, optimized by machine-learning training	***acc****_ap:* the acceleration in the anterior–posterior axis (orientation corrected) *Fs:* sampling frequency *GS:* array of gait sequences *ICs:* initial contacts [s] *Leg length* [cm] *Calibration factors* “*t*”, “*p*” (t = 0.85, p = foot size)	*output_sl*: a vector structure containing: *output_sl(N).start*: start of N-th GS [s] *output_sl(N).stop*: stop of N-th GS [s] *output_sl(N).slSec*: Estimated SL-per-second [m] of N-th GS *output_sl(N).slMean*: mean of SL [m] estimated for N-th GS *output_sl(N).slSTD*: std of SL [m] estimated for N-th GS *output_sl(N).distanc*e: covered distance [m] of N-th GS *output_sl(N).warnMes*: shows if there is a warning for N-th GS (1 means yes)	Matlab®	Zijlstra and Hof [47] Zijlstra and Zijlstra [48] Weinberg [51] Soltani, Aminian [17]

*Gait sequence detection* (*GSD*) This metric identifies sections of the raw signal which correspond to walking/gait. Three algorithms were selected: GSD_A_ [40], GSD_B_ [16] and GSD_C_ [41].

*Initial contact detection* (*ICD*) This metric detects the foot initial contact within each gait sequence. Four algorithms were selected: ICD_A_ [16, 41, 42], ICD_B_ [44], ICD_C_ [16, 41, 42] and ICD_D_ [45].

*Cadence estimation* (*CAD*) This metric identifies strides as a cyclic pattern from which cadence [number of steps within a minute (min)] is estimated in each walking bout [17]. Three algorithms were selected: CAD_A_ [41, 42, 44], CAD_B_ [16, 46] and CAD_C_ [17, 45]. Cadence (steps/min) was derived from identified strides as follows:

1 $${{\varvec{C}}{\varvec{a}}{\varvec{d}}{\varvec{e}}{\varvec{n}}{\varvec{c}}{\varvec{e}}}_{{\varvec{i}}}={StrideFrequency}_{i}*2,$$

where $i=1,\dots , n$ are the different walking bouts and Stride Frequency is evaluated as:

2 $$\user2{Stride\; Frequency}_{{\varvec{i}}} = \frac{{\mathop \sum \nolimits_{k = 1}^{{n\_STRIDE_{i} }} \left( {{\raise0.7ex\hbox{${60}$} \!\mathord{\left/ {\vphantom {{60} {STRIDE d_{k} }}}\right.\kern-0pt} \!\lower0.7ex\hbox{${STRIDE d_{k} }$}}} \right)}}{{n\_STRIDE_{i} }},$$

where $i=1,\dots , n$ are the different walking bouts, ${n\_STRIDE}_{i}$ is the number of strides (including right and left steps) in the relevant $i$ – walking bout, ${STRIDE d}_{k}$ is the duration [seconds] of the k – stride in the relevant $i-$ walking bout

*Stride length estimation* (*SL*) This metric quantifies stride length, evaluated as the distance between two non-consecutive initial contacts. Four algorithms were selected based either on biomechanical or machine-learning models: SL_A_ [47, 48], SL_B_ [47, 48], SL_C_ [49, 50] and SL_D_ [17, 51].

### Data and statistical analyses for validation and ranking of algorithms.

All calculations and statistical analysis were performed using Matlab® R2021a (Mathworks, Natick, MA).

#### Performance measures to describe and establish algorithm validity

To ensure objective comparison between systems (INDIP and wearable device), walking bouts detected by the INDIP were given as a standardized input to all algorithms except for gait sequence detection where the full wearable device recording was given as input. A walking bout was defined as a walking sequence containing at least two consecutive strides of both feet (e.g., R–L–R–L–R–L or L–R–L–R–L–R, with R/L being the right/left foot contact with the ground) [18]. Criteria for inclusion of a stride were: (a) duration of 0.2–3 s, and (b) a minimum length of 0.15 m. A resting period/break of 3 s or more identified consecutive walking bouts [18], thus each walking bout could include resting periods/breaks ≤ 3 s. Each metric was determined by the algorithms implemented on the single wearable device and by the INDIP.

Algorithm validation was established independently for each cohort by comparing digital mobility outcomes obtained from the selected algorithms applied to the wearable device with those from the INDIP using the following set of performance measures to describe and establish validity:

3 $${\text{Accuracy}} = \frac{TP + TN}{{TN + TP + FN + FP}}$$

4 $${\text{Sensitivity}} = \frac{TP}{{TP + FN}}$$

5 $${\text{Specificity}} = \frac{TN}{{TN + FP}}$$

6 $${\text{Positive\; Predictive\; Value}} = \frac{TP}{{TP + FP}}$$

where TP = True Positive events, TN = True Negative events, FP: False Positive events, FN: False Negative events.

- Intra class correlation coefficient (ICC_(2,1)_) [52] was calculated to assess the association between the digital mobility outcomes of the two systems using all walking bouts collected from each cohort separately. Based on ICC estimates, values less than 0.5, between 0.5 and 0.75, between 0.75 and 0.9, and greater than 0.9 were deemed to be indicative of poor, moderate, good, and excellent agreement, respectively [53].
- Absolute agreement was assessed by quantifying (i) absolute error, (ii) bias, and (iii) Limits of Agreement [54] between the wearable device and reference system digital mobility outcomes calculated for each walking bout.
- Relative errors between the wearable device and INDIP digital mobility outcomes were determined for each walking bout.

Mean and 95% confidence intervals of all digital mobility outcomes were evaluated at a cohort level (i.e., quantified using all walking bouts across all participants belonging to that specific cohort). Subsets of relevant measures were then used for the different digital mobility outcomes and evaluated as detailed below.

**Fig. 1:**
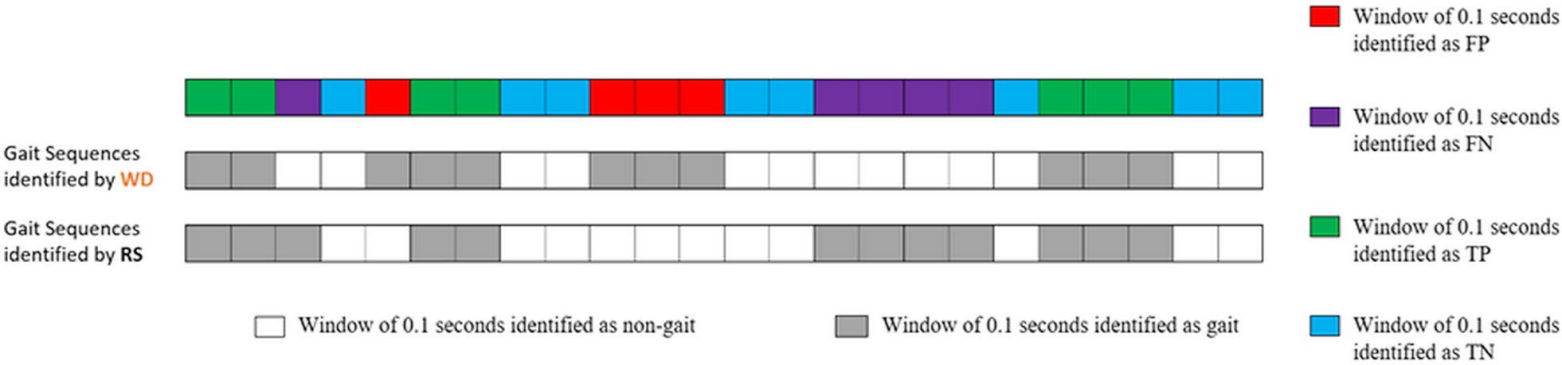
Example of identification of False Positive (FP), False Negative (FN), True Positive (TP) and True Negative (TN) for the Gait Sequence Detection (GSD) algorithms, of each window (0.1 s). Events classified from the comparison of each individual window between the INDIP reference system (RS) and the single wearable device (WD) for the detection of gait sequences. Each window of the WD and RS outputs are depicted as a rectangle, where white rectangles represent windows of non-gait sequences, and grey rectangles denote windows of a detected gait sequence

For gait sequence detection algorithms, each window of 0.1 s from the complete 2.5-h recording was classified (see Fig. 1) as either true positive, false positive, true negative or false negative and accuracy, sensitivity, specificity, positive predictive value were calculated. These measures were evaluated for each 2.5-h assessment. In addition, absolute errors and ICC_(2,1)_ for the total accumulated duration of all gait sequences identified in a 2.5-h recording was assessed and compared between the two systems, for each participant.

**Fig. 2:**
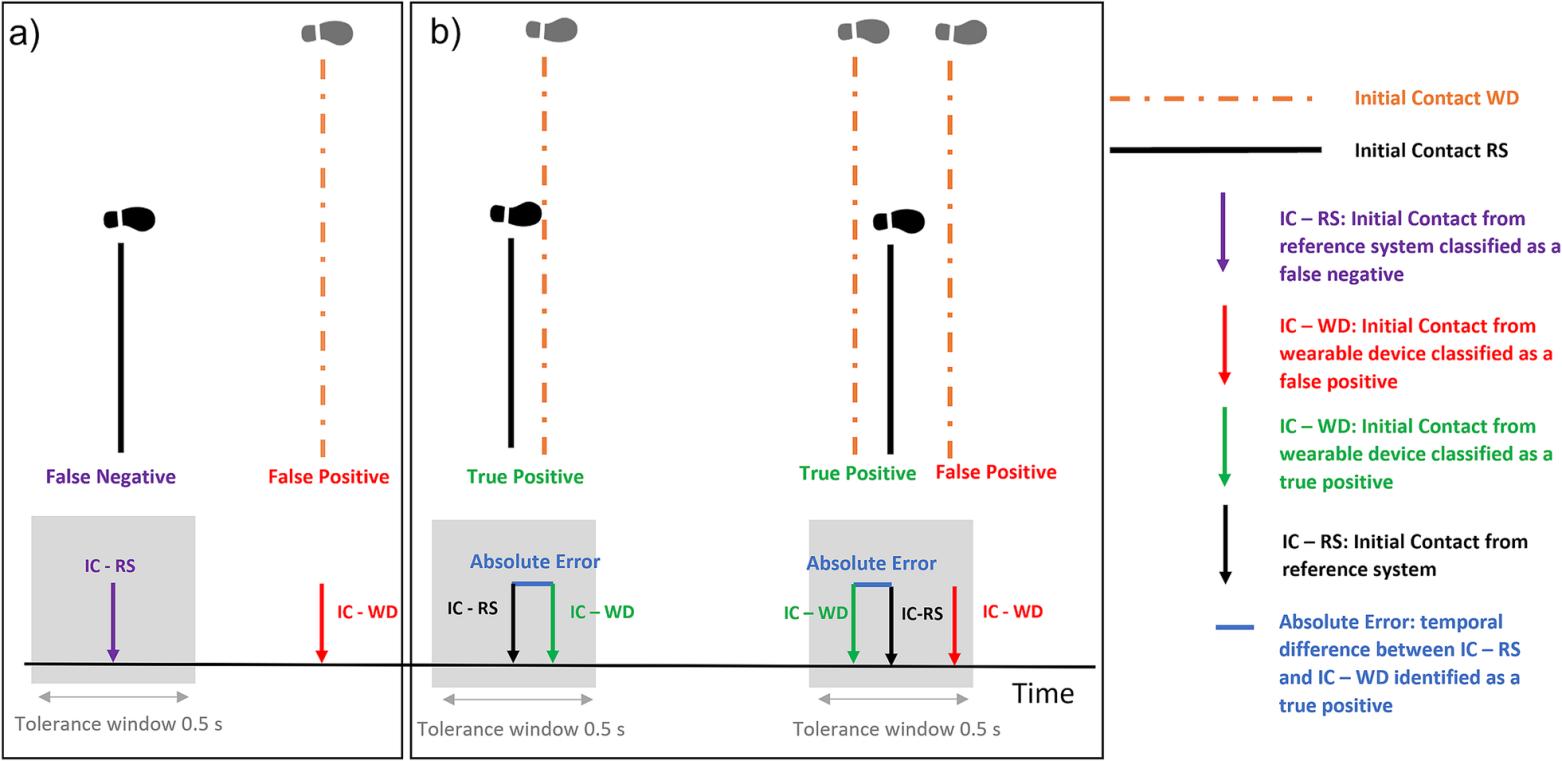
Example of performance analysis for initial contact detection (ICD) algorithms. The figure shows Initial contacts events identified by the reference system (IC-RS, depicted in black solid line) and initial contacts events identified by the single wearable device (IC-WD, depicted in orange dotted line). False Negatives, False Positive and True Positive events are defined with respect to the selected temporal tolerance window of 0.5 s (in grey) centred around the IC-RS. **a** Shows the identification of False Negative events (i.e., initial contact identified by the reference system but not identified by the single wearable device within the tolerance window) and False Positive events (i.e., initial contact wrongly identified by the single wearable device because although identified, it is outside the tolerance window). **b** Shows the identification of True Positives events (i.e., initial contact events correctly identified by the single wearable device) and example of other cases for identification of False Positive events (i.e., initial contact wrongly identified by the single wearable device). Note that the initial contact event, identified by the single wearable device, nearest to the true event (identified by the INDIP) will be considered a True Positive, and the rest of the identified events, False Positives. The figure also shows in blue how absolute errors are calculated only from True Positive events

In the case of initial contact detection, we defined each initial contact event within a walking bout as a true positive, false positive and false negative by comparing the initial contact events detected by the wearable device to the events detected by the INDIP within a tolerance window of 0.5 s (centred around the event identified by the INDIP, see Fig. 2), representative of a step duration [55]. This approach has been previously used and was adopted to take into account the potential mismatch on the event time between the INDIP and the wearable device [56]. To assess initial contact detection, true negative events were not evaluated, since true negative would correspond to all non-initial contact events identified as such by both systems.

For initial contact detection, we utilised the following measures: sensitivity, positive predictive values, absolute errors (which were estimated for each true positive initial contact (see Fig. 2)) and relative error (estimated by dividing all absolute errors, within a walking bout, by the average step duration estimated by the INDIP [55]).

For cadence and stride length algorithms, the measures used were: relative errors, absolute errors and ICC_(2,1)_.

#### Ranking algorithms using performance measures

A simplified version of the ranking methodology described in Bonci et al. [24] was applied to compare algorithm performance using a decision matrix. This was based on the weighted combination of performance measures described above assessing agreement between the single wearable device and the INDIP system (classified as benefit or cost). Performance measures considered as benefits were: accuracy, sensitivity, specificity, positive predictive value and ICC_(2,1)_ [52]. Performance measures considered as costs were absolute and relative errors. Each measure was weighted based on its relative importance to the algorithm’s validity assessment (see Bonci et al. [24] and Additional file 1 for further detail regarding the specific performance measures and assigned weights for gait sequence detection, initial contact detection, cadence and stride length algorithms). This information was combined to determine a performance index (0 = worst, 1 = best), calculated as a weighted mean of the selected benefit and/or cost analysis, which was subsequently used to compare and rank the algorithm performances, and thus, to select the top performing algorithms for each cohort independently.

#### Influence of walking speed and walking duration on the algorithms’ performance

The performance of initial contact detection, cadence and stride length top-selected algorithms was then assessed considering the impact that walking bout walking speed values (calculated as the average stride speed by the INDIP system) and walking bout durations had on the relative error of each digital mobility outcome (i.e., step duration, cadence and stride length). Specifically, median relative errors for each digital mobility outcome were quantified evaluating all the walking bouts characterized by specific walking speed and walking bout duration ranges; including errors observed in consecutive walking speed windows of 0.05 m/s [57] and in consecutive walking bout duration windows of 2 s. For each digital mobility outcome, the resulting median errors were then employed in a best-fit approach to determine their association between the relative errors and walking speed or walking bout duration, respectively. In the best-fit approach, median error values were also weighted according to the relevant number of observations in a given window with respect to the total number of observations.

## Results

Participant clinical and demographic characteristics per cohort are presented in Table 2.

**Table 2:** Demographic and clinical characteristics of the participants

Characteristic (unit of measure) or [range]	HA (n = 20)	CHF (n = 12)	COPD (n = 17)	MS (n = 20)	PD (n = 20)	PFF (n = 19)
Number of walking bouts included in the analyses (n)	1343	416	1031	795	681	684
Age (years)	71.7 ± 5.8	69.1 ± 11.7	69.4 ± 9.1	48.7 ± 9.7	69.8 ± 7.2	80.0 ± 8.5
Height (cm)	1.66 ± 0.10	1.74 ± 0.10	1.69 ± 0.07	1.71 ± 0.13	1.73 ± 0.07	1.69 ± 0.08
Weight (kg)	75.1 ± 11.8	84.5 ± 16.8	73.7 ± 14.2	84.0 ± 22.9	78.2 ± 14.4	68.4 ± 16.0
Gender: % females [females n, males n]	45% [9, 11]	33% [4, 8]	47% [8, 9]	45% [9, 11]	20% [4, 16]	58% [8, 11]
WS during the 2.5-h assessment (mean and [range]) (m/s)	0.59 [0.12, 1.63]	0.72 [0.14, 1.46]	0.60 [0.11, 1.36]	0.58 [0.15, 1.60]	0.60 [0.10, 1.44]	0.54 [0.14, 1.29]
Walking Aid use: % of users [n]	5% [1]	25% [3]	6% [1]	25% [5]	30% [6]	68% [13]
MoCA [0–30]	27.7 ± 2.6	27.1 ± 2.9	24.6 ± 3.4	26.7 ± 3.1	24.6 ± 4.0	24.1 ± 4.2
LLFDI [0–100]	73.53 ± 14.22	67.29 ± 21.35	59.07 ± 7.96	57.34 ± 10.66	60.26 ± 12.51	52.59 ± 16.61
Hoehn & Yahr stage (n)					H&Y I: 4 H&Y II: 11 H&Y III: 5	
MDS-UPDRS III [0–132]					28.4 ± 13.6	
EDSS [0–6]				3.5 ± 1.7		
SPPB [0–12]						6.2 ± 3.9
CAT Score [0–40]			16.6 ± 8.9			
FEV1 (litres)			1.6 ± 0.6			
6MWT distance (m)		370.7 ± 115.6	357.6 ± 88.5			
KCCQ-12 Score [0–100]		80.5 ± 20.2				

Values are presented as mean ± standard deviation, unless otherwise stated

*CAT* chronic obstructive pulmonary disease (COPD) Assessment Test; *EDSS* Expanded Disability Status Scale; *FEV1* Forced Expiratory Volume in 1 Second; *KCCQ-12* Kansas City Cardiomyopathy Questionnaire-12; *LLFDI* Late Life Function and Disability Instrument; *MDS-UPDRS III* Movement Disorder Society Unified Parkinson’s Disease Rating Scale Part III; *MoCA* Montreal Cognitive Assessment; *SPPB* Short Physical Performance Battery; *6MWT* 6 Minute Walking Test; *HA* healthy adults; *PD* Parkinson’s disease; *MS* multiple sclerosis; *COPD* chronic obstructive pulmonary disease; *CHF* congestive heart failure; *PFF* proximal femoral fracture

The cohorts covered a wide range of mobility levels: the walking speed measured by the INDIP system during the 2.5-h assessment ranged from an average of 0.54 m/s (proximal femoral fracture) to 0.72 m/s (congestive heart failure), with a minimum measured walking speed of 0.10 m/s (in Parkinson’s disease) and a maximum of 1.63 m/s (in healthy older adults) (Table 2).

Nine participants (8%: three with chronic heart failure, two with multiple sclerosis, one with Parkinson’s disease and three proximal femoral fracture participants) were excluded from subsequent analysis due to data unavailability.

### Gait sequence detection

#### Performance measures and ranking

We report in Table 3 the gait sequence detection algorithms main peformance measures (All performance measures are considered for the evaluation of the performance index are shown in the Additional file 1: Table).

**Table 3:** Gait sequence detection (GSD) performance measures; gait sequence total duration obtained from the INDIP and the single wearable device, absolute error, bias and limits of agreement (LoA) and intra class correlation (ICC_(2,1)_) for comparison between systems, and overall performance index for the GSD algorithms. Values are expressed as mean and 95% confidence intervals (CI) for each cohort. In italic and boldface recommended algorithms. Underlined performance index indicates top-ranked algorithm for the specific cohort of that row

Cohort	Gait sequence detection				Gait sequence total duration					Performance index
	Sensitivity	Positive predictive value	Accuracy	Specificity	INDIP mean and CI [s]	Single wearable device mean and CI [s]	Bias and LoA [s]	Absolute error [s]	ICC (2,1)	
GSD_A_										
*HA*	**0.84 [0.77, 0.92]**	**0.81 [0.75, 0.88]**	**0.94 [0.93, 0.96]**	**0.96 [0.95, 0.97]**	**1640.0 [1273.4, 2006.6]**	**1708.5 [1316.3, 2100.7]**	**68.5 [− 710.6, 847.5]**	**215.7 [57.7, 373.7]**	**0.88 [0.73, 0.95]**	**0.819**
*CHF*	**0.85 [0.68, 1.01]**	**0.81 [0.72, 0.90]**	**0.95 [0.92, 0.98]**	**0.96 [0.94, 0.99]**	**1545.4 [421.1, 2669.6]**	**1595.4 [395.2, 2795.6]**	**50.0 [− 689.5, 789.5]**	**242.0 [39.6, 444.4]**	**0.98 [0.91, 0.99]**	**0.853**
*COPD*	**0.85 [0.81, 0.88]**	**0.75 [0.68, 0.82]**	**0.95 [0.93, 0.97]**	**0.96 [0.95, 0.98]**	**1004.8 [829.2, 1180.4]**	**1151.6 [955.7, 1347.6]**	**146.8 [− 369.1, 662.8]**	**176.5 [51.5, 301.5]**	**0.68 [0.32, 0.87]**	**0.822**
*MS*	**0.92 [0.87, 0.97]**	**0.75 [0.66, 0.84]**	**0.95 [0.92, 0.98]**	**0.95 [0.92, 0.98]**	**1119.5 [736.9, 1502.2]**	**1452.0 [990.4, 1913.6]**	**332.5 [− 883.9, 1548.8]**	**358.5 [57.7, 659.4]**	**0.68 [0.34, 0.87]**	**0.735**
*PD*	**0.86 [0.78, 0.94]**	**0.81 [0.71, 0.91]**	**0.96 [0.95, 0.98]**	**0.97 [0.96, 0.99]**	**1121.8 [626.5, 1617.1]**	**1177.3 [656.2, 1698.5]**	**55.6 [− 402.4, 513.6]**	**139.1 [45.8, 232.4]**	**0.98 [0.94, 0.99]**	**0.852**
PFF	0.71 [0.54, 0.89]	0.74 [0.61, 0.88]	0.95 [0.92, 0.97]	0.97 [0.96, 0.99]	954.4 [599.5, 1309.4]	975.7 [589.8, 1361.6]	21.3 [− 365.3, 407.9]	144.4 [72.1, 216.7]	0.96 [0.89, 0.99]	0.770
GSD_B_										
HA	0.86 [0.82, 0.90]	0.85 [0.79, 0.92]	0.95 [0.94, 0.96]	0.97 [0.96, 0.98]	1645.8 [1258.1, 2033.5]	1657.4 [1283.5, 2031.3]	11.6 [− 592.4, 615.6]	170.5 [48.1, 292.8]	0.93 [0.83, 0.97]	0.727
CHF	0.91 [0.88, 0.94]	0.82 [0.71, 0.92]	0.96 [0.94, 0.98]	0.96 [0.94, 0.99]	1545.4 [421.1, 2669.6]	1689.6 [557.8, 2821.5]	144.3 [− 331.8, 620.4]	176.1 [19.8, 332.3]	0.99 [0.95, 1.00]	0.792
COPD	0.84 [0.81, 0.88]	0.80 [0.77, 0.82]	0.96 [0.95, 0.97]	0.97 [0.97, 0.98]	1004.8 [829.2, 1180.4]	1058.4 [865.5, 1251.4]	53.6 [− 120.3, 227.6]	71.9 [34.0, 109.8]	0.96 [0.90, 0.99]	0.814
**MS**	*0.91 [0.87, 0.95]*	*0.75 [0.65, 0.85]*	*0.95 [0.92, 0.98]*	*0.95 [0.92, 0.98]*	*1119.5 [736.9, 1502.2]*	*1416.3 [947.8, 1884.8]*	*296.8 [− 911.6, 1505.2]*	*323.1 [23.5, 622.6]*	*0.70 [0.38, 0.88]*	*0.655*
**PD**	*0.84 [0.76, 0.92]*	*0.83 [0.73, 0.93]*	*0.96 [0.95, 0.97]*	*0.98 [0.96, 0.99]*	*1121.8 [626.5, 1617.1]*	*1145.9 [629.1, 1662.6]*	*24.1 [− 450.9, 499.0]*	*167.9 [85.1, 250.8]*	*0.97 [0.94, 0.99]*	*0.726*
*PFF*	**0.73 [0.60, 0.86]**	**0.84 [0.76, 0.93]**	**0.96 [0.95, 0.98]**	**0.98 [0.97, 0.99]**	**911.7 [569.4, 1254.0]**	**861.1 [542.8, 1179.3]**	**− 50.7 [− 445.9, 344.6]**	**141.4 [62.2, 220.6]**	**0.95 [0.86, 0.98]**	**0.771**
GSD_C_										
HA	0.77 [0.71, 0.83]	0.91 [0.85, 0.98]	0.95 [0.94, 0.96]	0.99 [0.98, 1.00]	1645.8 [1258.1, 2033.5]	1409.3 [1034.8, 1783.7]	− 236.5 [− 848.7, 375.7]	323.6 [220.1, 427.1]	0.88 [0.73, 0.95]	0.722
CHF	0.84 [0.79, 0.88]	0.89 [0.82, 0.96]	0.97 [0.95, 0.98]	0.98 [0.97, 0.99]	1545.4 [421.1, 2669.6]	1466.6 [359.8, 2573.4]	− 78.7 [− 309.9, 152.4]	113.1 [55.1, 171.1]	1.00 [0.99, 1.00]	0.811
COPD	0.73 [0.68, 0.78]	0.89 [0.87, 0.91]	0.96 [0.95, 0.97]	0.99 [0.99, 0.99]	1004.8 [829.2, 1180.4]	825.2 [664.4, 986.1]	− 179.6 [− 420.7, 61.6]	179.6 [116.3, 242.8]	0.80 [0.53, 0.92]	0.776
MS	0.83 [0.77, 0.90]	0.83 [0.73, 0.93]	0.96 [0.93, 0.98]	0.97 [0.94, 1.00]	1119.5 [736.9, 1502.2]	1223.3 [794.0, 1652.5]	103.8 [− 1100.0, 1307.5]	246.9 [− 36.2, 530.0]	0.72 [0.41, 0.89]	0.693
PD	0.76 [0.65, 0.87]	0.87 [0.76, 0.98]	0.96 [0.95, 0.97]	0.99 [0.98, 1.00]	1121.8 [626.5, 1617.1]	1000.4 [514.3, 1486.5]	− 121.4 [− 579.4, 336.5]	192.3 [107.3, 277.3]	0.97 [0.92, 0.99]	0.726
PFF	0.60 [0.45, 0.75]	0.89 [0.77, 1.01]	0.94 [0.91, 0.97]	0.99 [0.99, 1.00]	954.4 [599.5, 1309.4]	697.3 [439.9, 954.8]	− 257.1 [− 823.6, 309.4]	272.4 [120.9, 423.9]	0.78 [0.47, 0.92]	0.687

*HA* healthy adults; *PD* Parkinson’s disease; *MS* multiple sclerosis; *COPD* chronic obstructive pulmonary disease; *CHF* congestive heart failure; *PFF* proximal femoral fracture; *CI* confidence intervals, *LoA* limits of agreement, *ICC* intra class correlation

Across all cohorts, performance measures for the three gait sequence detection algorithms were good to excellent (sensitivity ranged between 0.60 and 0.92, specificity between 0.95 and 0.99, accuracy between 0.94 and 0.97 and positive predictive value between 0.74 and 0.91 [41] (Table 3, Additional file 1: Table). The lowest sensitivity was observed for the most impaired cohort (proximal femoral fracture) for all algorithms.

The absolute error between the wearable device and the INDIP for the total accumulated duration of the detected gait sequences ranged from 71.9 to 358.5 s across the three algorithms which was approximately from 7 to 32% of the total duration estimated by the INDIP. Overall, except for the proximal femoral fracture cohort, GSD_A_ and GSD_B_ overestimated the total gait sequence duration, whereas GSD_C_ underestimated it. The ICC_(2,1)_ ranged from 0.68 to 1.00, with the lowest ICC_(2,1)_ found for the multiple sclerosis cohort, in line with the largest disagreement, based on the largest limits of agreement [54], among all cohorts and the three algorithms.

Algorithm **GSD**_**A**_ presented the overall best performance index for healthy older adults (0.819), congestive heart failure (0.853), chronic obstructive pulmonary disease (0.822), multiple sclerosis (0.735) and Parkinson’s disease (0.852) cohorts (see Additional file 1). Algorithm **GSD**_**B**_ presented the highest performance indexes for the proximal femoral fracture cohort (0.771) and similar good performances for multiple sclerosis (0.655) and Parkinson’s disease (0.726).

### Initial contact detection

#### Performance measures and ranking

Table 4 presents performance measures of initial contact detection algorithms, which were very similar for the four algorithms. Across algorithms and cohorts, sensitivity ranged from 0.76 to 0.83 and positive predictive values from 0.81 to 0.93, whilst relative errors ranged from 7.6 to 21.2%.

**Table 4:** Initial contact detection (ICD) performance measures. Sensitivity, positive predictive value, absolute and relative errors, and overall performance index for the ICD algorithms. Values are expressed as mean and 95% confidence intervals (CI) for each cohort. In italic face: recommended algorithms. Underlined performance index indicates top-ranked algorithm for the specific cohort of that row

Cohort	Initial contact detection				
	Sensitivity	Positive predictive value	Absolute error [s]	Relative error [%]	Performance index
ICD_A_					
*HA*	**0.80 [0.79, 0.81]**	**0.91 [0.90, 0.92]**	**0.06 [0.05, 0.06]**	**8.0 [7.7, 8.3]**	**0.804**
*CHF*	**0.79 [0.77, 0.80]**	**0.89 [0.88, 0.91]**	**0.07 [0.06, 0.07]**	**10.9 [10.1, 11.8]**	**0.771**
*COPD*	**0.81 [0.81, 0.82]**	**0.93 [0.92, 0.94]**	**0.07 [0.06, 0.07]**	**9.1 [8.8, 9.3]**	**0.790**
*MS*	**0.80 [0.79, 0.82]**	**0.92 [0.91, 0.93]**	**0.06 [0.06, 0.06]**	**8.4 [8.1, 8.7]**	**0.805**
*PD*	**0.79 [0.77, 0.80]**	**0.90 [0.89, 0.91]**	**0.06 [0.06, 0.06]**	**8.3 [7.9, 8.6]**	**0.798**
*PFF*	**0.80 [0.78, 0.81]**	**0.89 [0.88, 0.90]**	**0.05 [0.05, 0.06]**	**7.6 [7.3, 7.9]**	**0.818**
ICD_B_					
HA	0.80 [0.79, 0.81]	0.86 [0.85, 0.87]	0.13 [0.13, 0.13]	18.0 [17.8, 18.3]	0.641
CHF	0.80 [0.78, 0.81]	0.88 [0.86, 0.90]	0.13 [0.13, 0.14]	19.9 [19.3, 20.4]	0.641
COPD	0.78 [0.77, 0.79]	0.85 [0.84, 0.86]	0.15 [0.15, 0.15]	21.2 [20.9, 21.5]	0.590
MS	0.75 [0.73, 0.77]	0.81 [0.79, 0.83]	0.14 [0.14, 0.15]	19.8 [19.3, 20.4]	0.595
PD	0.80 [0.78, 0.81]	0.86 [0.84, 0.88]	0.14 [0.14, 0.14]	19.2 [18.9, 19.6]	0.620
PFF	0.80 [0.79, 0.82]	0.86 [0.85, 0.88]	0.13 [0.13, 0.13]	17.9 [17.5, 18.2]	0.641
ICD_C_					
HA	0.82 [0.81, 0.83]	0.86 [0.85, 0.88]	0.06 [0.06, 0.07]	8.9 [8.6, 9.2]	0.795
CHF	0.81 [0.80, 0.82]	0.87 [0.85, 0.88]	0.07 [0.07, 0.08]	11.1 [10.2, 11.9]	0.771
COPD	0.83 [0.82, 0.83]	0.90 [0.89, 0.91]	0.07 [0.07, 0.07]	9.7 [9.4, 9.9]	0.786
MS	0.82 [0.81, 0.84]	0.89 [0.87, 0.90]	0.07 [0.06, 0.07]	9.2 [8.8, 9.6]	0.783
PD	0.80 [0.78, 0.81]	0.84 [0.82, 0.86]	0.07 [0.07, 0.07]	9.4 [9.0, 9.8]	0.766
PFF	0.80 [0.79, 0.82]	0.81 [0.79, 0.82]	0.07 [0.07, 0.07]	9.6 [9.2, 10.0]	0.759
ICD_D_					
HA	0.78 [0.77, 0.79]	0.88 [0.87, 0.89]	0.10 [0.10, 0.10]	14.1 [13.8, 14.5]	0.705
CHF	0.79 [0.78, 0.81]	0.92 [0.91, 0.93]	0.09 [0.09, 0.10]	13.8 [13.4, 14.3]	0.735
COPD	0.76 [0.75, 0.77]	0.89 [0.88, 0.90]	0.11 [0.11, 0.12]	15.9 [15.5, 16.3]	0.680
MS	0.78 [0.77, 0.80]	0.89 [0.88, 0.91]	0.10 [0.10, 0.11]	14.4 [13.9, 15.0]	0.706
PD	0.76 [0.75, 0.78]	0.86 [0.84, 0.87]	0.11 [0.11, 0.11]	15.4 [15.0, 15.8]	0.674
PFF	0.80 [0.78, 0.81]	0.86 [0.85, 0.88]	0.10 [0.09, 0.10]	13.6 [13.1, 14.1]	0.706

*HA* healthy adults; *PD* Parkinson’s disease; *MS* multiple sclerosis; *COPD* chronic obstructive pulmonary disease; *CHF* congestive heart failure; *PFF* proximal femoral fracture; *LoA* limits of agreement

Algorithm **ICD**_**A**_ presented the highest overall performance index across all cohorts: healthy older adults (0.804), congestive heart failure (0.771), chronic obstructive pulmonary disease (0.790), multiple sclerosis (0.805), Parkinson’s disease (0.798) and proximal femoral fracture (0.818) reflecting the lowest absolute and relative errors, highest sensitivity, and positive predictive values.

#### Effect of walking speed and bout duration

**Fig. 3:**
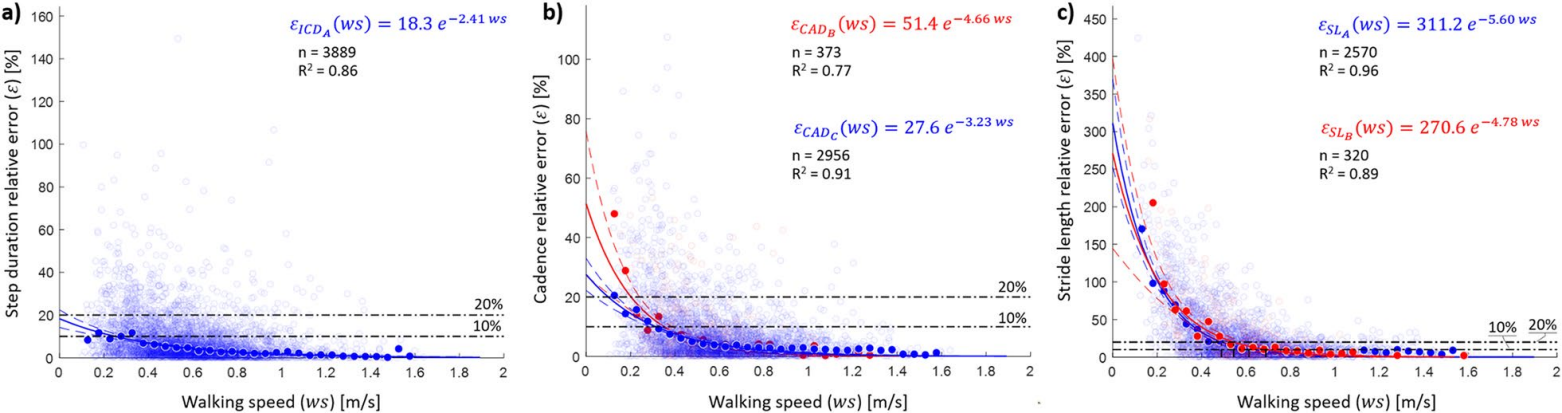
Effect of walking speed on the relative errors in **a** step duration, **b** CAD and **c** step length estimations. The empty shaded circles (n) represent the relative error for each walking bout when the selected algorithms are used (ICD_A_ for all cohorts in blue; CAD_B_ for the congestive heart failure (CHF), chronic obstructive pulmonary disease (COPD), healthy adults (HA), multiple sclerosis (MS) and Parkinson’s disease (PD) cohorts in red; CAD_C_ for the proximal femoral fracture (PFF) cohort in blue; SL_A_ for CHF, COPD, HA, PFF and PD in blue; and SL_B_ for MS in red). The filled circles indicate the median relative errors quantified in consecutive walking speed (*ws*) windows of 0.05 m/s. The sizes of the filled circles have been calculated as the ratio between the number of empty shaded circles in a given walking speed window and the total number of empty shaded circles (i.e., all observations). The obtained exponential curves (represented in blue and red continuous lines) and equations are the result of a best-fit approach used to determine the association between walking speed and the calculated median errors; R^2^ values are also shown. The horizontal black dash-dotted lines visually indicate the relative error thresholds of 10 and 20%

**Fig. 4:**
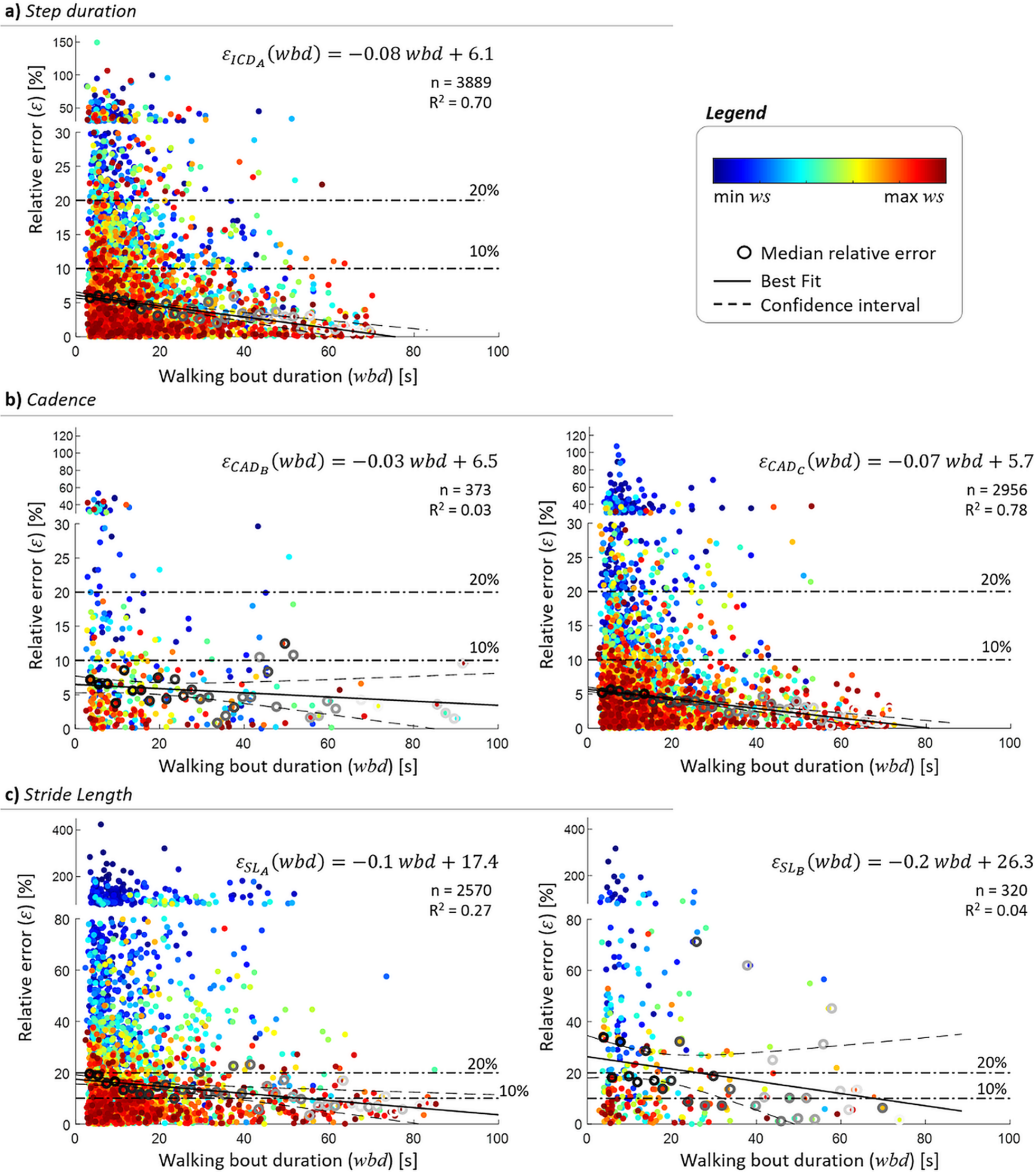
Effect of walking bout duration on the relative errors ($\varepsilon$) in **a** step duration, **b** cadence, and **c** step length estimations. For each parameter and the selected algorithms (ICD_A_ for all cohorts; CAD_B_ for the congestive heart failure (CHF), chronic obstructive pulmonary disease (COPD), healthy adults (HA), multiple sclerosis (MS) and Parkinson’s disease (PD) cohorts; CAD_C_ for the proximal femoral fracture (PFF) cohort; SL_A_ for CHF, COPD, HA, PFF and PD; and SL_B_ for MS), the individual filled circles (n) represent the relative error for each WB, colour coded according to the walking speed measured for that specific bout. The empty grey circles indicate the median relative errors quantified in subsequent separate walking bout duration (*wbd*) windows of 2 s. Grey intensity represents the weight associated to the relevant observation, calculated as the ratio between the number of points in a given walking bout duration window and the total number of points, in the best-fit approach; a darker grey represents a larger weight. The black continuous lines and equations are the result of a best-fit approach used to determine the association between walking bout duration and the median errors; R^2^ values are also shown. The horizontal black dash-dotted lines visually indicate the relative error thresholds of 10% and 20%. For a clear visualization of the results, 95% of the walking bout duration have been reported

Relative errors for step duration, as extracted from the initial contacts, decreased with walking speed (R^2^ = 0.86), with errors lower than 10% reached for walking speeds > 0.25 m/s (Fig. 3a) [58]. Any value of walking bout duration showed median errors lower than 10%, but an overall error decrease was observed when the walking bout duration increased (R^2^ = 0.70, Fig. 4a). Overall, higher errors (> 50%) were observed only in the 0.9% of the detected walking bouts; these bouts were characterised by a short duration (8.37 ± 4.71 s) and slow walking speed (0.44 ± 0.24 m/s).

### Cadence estimation

#### Performance measures and ranking

Performance measures of the cadence algorithms are presented in Table 5, reflecting a slight (4.6–7.2 steps/min) overestimation of cadence by the wearable device with respect to INDIP for all the cohorts with algorithms CAD_B_ and CAD_C_ (except for proximal femoral fracture with CAD_C_, in which case there is a misestimation). The absolute error ranged from 5.2 to 9.3 steps/min, the relative error between 6.6% to 11.8% and ICC_(2,1)_ ranged from 0.44 to 0.82 across the three algorithms.

**Table 5:** Cadence (CAD) estimation performance measures. Cadence obtained from the INDIP and the single wearable device, bias, limits of agreement (LoA) and intra class correlation (ICC(2,1)) for comparison between systems, and overall performance index for the CAD algorithms. In italic and boldface recommended algorithms. Underlined performance index indicates top-ranked algorithm for the specific cohort of that row

Cohort	Cadence						
	INDIP mean and CI [steps/min]	Single wearable device mean and CI [steps/min]	Bias and LoA [steps/min]	Absolute error [steps/min]	Relative error [%]	ICC (2, 1)	Performance index
CAD_A_							
HA	86.0 [85.1, 87.0]	85.6 [84.4, 86.8]	− 0.4 [− 27.0, 26.2]	8.6 [7.9, 9.3]	10.5 [9.6, 11.5]	0.65 [0.61, 0.69]	0.538
CHF	91.2 [89.8, 92.5]	89.8 [88.2, 91.5]	− 1.4 [− 30.7, 28.0]	9.3 [8.2, 10.3]	10.6 [9.5, 11.8]	0.63 [0.57, 0.68]	0.517
COPD	84.6 [83.7, 85.4]	86.3 [85.2, 87.3]	1.7 [− 17.5, 20.8]	6.7 [6.2, 7.2]	8.3 [7.6, 9.0]	0.72 [0.68, 0.75]	0.644
MS	84.6 [83.1, 86.1]	85.7 [83.9, 87.5]	1.1 [− 22.8, 25.0]	8.2 [7.3, 9.2]	10.1 [8.9, 11.3]	0.71 [0.65, 0.75]	0.594
PD	84.8 [83.4, 86.1]	87.0 [85.4, 88.6]	2.2 [− 24.9, 29.4]	9.1 [8.1, 10.1]	11.8 [10.4, 13.2]	0.62 [0.56, 0.67]	0.520
PFF	85.0 [83.4, 86.5]	81.6 [79.9, 83.4]	− 3.3 [− 30.6, 23.9]	8.9 [7.7, 10.0]	10.4 [9.1, 11.7]	0.60 [0.54, 0.67]	0.529
CAD_B_							
HA	86.0 [85.1, 87.0]	89.8 [88.9, 90.6]	3.7 [− 16.3, 23.8]	7.1 [6.6, 7.7]	9.1 [8.3, 9.9]	0.69 [0.65, 0.72]	0.585
CHF	91.2 [89.8, 92.5]	94.6 [93.5, 95.8]	3.5 [− 14.0, 21.0]	6.4 [5.8, 7.0]	7.7 [6.9, 8.5]	0.79 [0.76, 0.82]	0.654
COPD	84.6 [83.7, 85.4]	89.1 [88.3, 89.9]	4.5 [− 10.4, 19.4]	6.2 [5.7, 6.6]	7.9 [7.2, 8.6]	0.71 [0.67, 0.74]	0.660
**MS**	*84.6 [83.1, 86.1]*	*88.5 [87.1, 89.9]*	*3.9 [− 15.2, 23.0]*	*7.0 [6.2, 7.8]*	*9.0 [7.8, 10.2]*	*0.72 [0.66, 0.76]*	*0.633*
**PD**	*84.8 [83.4, 86.1]*	*89.5 [88.4, 90.7]*	*4.8 [− 14.3, 23.8]*	*7.2 [6.5, 8.0]*	*9.7 [8.5, 10.8]*	*0.69 [0.63, 0.73]*	*0.580*
*PFF*	**85.0 [83.4, 86.5]**	**86.2 [85.0, 87.5]**	**1.3 [− 20.9, 23.5]**	**7.2 [6.3, 8.1]**	**8.5 [7.5, 9.5]**	**0.66 [0.60, 0.71]**	**0.584**
CAD_C_							
*HA*	**86.0 [85.1, 87.0]**	**87.4 [86.5, 88.3]**	**1.4 [− 18.4, 21.2]**	**6.4 [5.9, 6.9]**	**8.1 [7.3, 8.8]**	**0.74 [0.70, 0.76]**	**0.653**
*CHF*	**91.2 [89.8, 92.5]**	**92.2 [90.9, 93.5]**	**1.0 [− 16.9, 19.0]**	**5.8 [5.1, 6.4]**	**6.6 [5.9, 7.3]**	**0.82 [0.79, 0.84]**	**0.720**
*COPD*	**84.6 [83.7, 85.4]**	**86.6 [85.8, 87.4]**	**2.0 [− 12.9, 17.0]**	**5.2 [4.8, 5.6]**	**6.6 [6.0, 7.2]**	**0.77 [0.73, 0.79]**	**0.693**
*MS*	**84.6 [83.1, 86.1]**	**85.3 [83.9, 86.8]**	**0.7 [− 18.8, 20.3]**	**6.7 [5.9, 7.5]**	**8.2 [7.2, 9.2]**	**0.75 [0.70, 0.79]**	**0.644**
*PD*	**84.8 [83.4, 86.1]**	**87.1 [85.9, 88.4]**	**2.3 [− 16.2, 20.9]**	**6.4 [5.7, 7.1]**	**8.2 [7.2, 9.2]**	**0.76 [0.71, 0.79]**	**0.653**
PFF	85.0 [83.4, 86.5]	82.0 [80.7, 83.3]	− 2.9 [− 31.5, 25.6]	8.3 [7.0, 9.5]	9.1 [7.9, 10.4]	0.44 [0.35, 0.51]	0.460

*HA* healthy adults; *PD* Parkinson’s disease; *MS* multiple sclerosis; *COPD* chronic obstructive pulmonary disease; *CHF* congestive heart failure; *PFF* proximal femoral fracture; *CI* confidence intervals, *LoA* limits of agreement, *ICC* intra class correlation

The highest absolute and relative errors, and the lowest ICC_(2,1)_ were found for the proximal femoral fracture cohort. **CAD**_**C**_ had the highest performance index for healthy older adults (0.653), congestive heart failure (0.720), chronic obstructive pulmonary disease (0.693), multiple sclerosis (0.644), Parkinson’s disease (0.653). **CAD**_**B**_ presented the best performances for proximal femoral fracture (0.584) showing the lowest absolute error (7.2 steps/min), closest largest limits of agreement (− 10.1 to 24.2 steps/min), lowest relative error (8.5%) and highest ICC_(2,1)_ (0.66). Overall good performances were also found for CAD_B_ for multiple sclerosis and Parkinson’s disease.

#### Effect of walking speed and bout duration

For both CAD_B_ and CAD_C_, as walking speed increased, the relative error decreased (Fig. 3b), with speeds above 0.3 m/s resulting in an error below a 10% threshold [58]. Generally, the highest errors were observed for the shortest and slowest bouts (Fig. 4b). The walking bouts with higher errors [> 50%, n = 25 (0.8%)] had a mean duration of 8.88 s (std: 5.97 s) and slow walking speed values (0.28 ± 0.09 m/s).

### Stride length estimation

#### Performance measures and ranking

Table 6 shows an overall overestimation of stride length by the wearable device with respect to the INDIP. The absolute error between the wearable device and the INDIP outcomes ranged from 0.15 to 0.33 m across all algorithms.

**Table 6:** Stride length (SL) estimation performance measures. Stride length obtained from the INDIP and the single wearable device, bias, limits of agreement (LoA) and intra class correlation (ICC(2,1)) for comparison between systems, and overall performance index for the SL algorithms. In boldface: recommended algorithms. Underlined performance index indicates top-ranked algorithm for the specific cohort of that row

Cohort	Stride length						
	INDIP mean and CI [m]	Single wearable device mean and CI [m]	Bias and LoA [m]	Absolute error [m]	Relative error [%]	ICC (2,1)	Performance index
SL_A_							
*HA*	**0.81 [0.79, 0.83]**	**0.93 [0.91, 0.94]**	**0.12 [− 0.24, 0.48]**	**0.15 [0.14, 0.17]**	**25.9 [23.2, 28.6]**	**0.58 [0.53, 0.63]**	**0.582**
*CHF*	**0.93 [0.90, 0.95]**	**1.00 [0.98, 1.02]**	**0.07 [− 0.34, 0.49]**	**0.16 [0.15, 0.18]**	**25.3 [22.4, 28.1]**	**0.70 [0.65, 0.74]**	**0.663**
*COPD*	**0.85 [0.83, 0.87]**	**1.03 [1.02, 1.05]**	**0.18 [− 0.20, 0.57]**	**0.21 [0.19, 0.22]**	**31.2 [28.0, 34.3]**	**0.28 [0.21, 0.36]**	**0.381**
MS	0.82 [0.79, 0.85]	1.02 [0.99, 1.04]	0.19 [− 0.22, 0.61]	0.21 [0.19, 0.24]	34.1 [29.3, 38.8]	0.41 [0.32, 0.50]	0.462
*PD*	**0.82 [0.79, 0.85]**	**0.93 [0.90, 0.95]**	**0.11 [− 0.31, 0.52]**	**0.17 [0.15, 0.18]**	**26.5 [23.0, 30.0]**	**0.60 [0.54, 0.66]**	**0.607**
*PFF*	**0.75 [0.73, 0.78]**	**0.86 [0.84, 0.87]**	**0.10 [− 0.32, 0.52]**	**0.17 [0.15, 0.19]**	**29.3 [25.2, 33.4]**	**0.36 [0.26, 0.46]**	**0.465**
SL_B_							
HA	0.81 [0.79, 0.83]	0.97 [0.95, 0.98]	0.16 [− 0.20, 0.52]	0.18 [0.17, 0.19]	29.6 [26.7, 32.5]	0.52 [0.47, 0.57]	0.546
CHF	0.93 [0.90, 0.95]	1.04 [1.02, 1.07]	0.12 [− 0.30, 0.53]	0.17 [0.16, 0.19]	27.4 [24.2, 30.5]	0.66 [0.61, 0.71]	0.604
COPD	0.85 [0.83, 0.87]	1.08 [1.06, 1.09]	0.23 [− 0.16, 0.62]	0.24 [0.23, 0.25]	35.8 [32.5, 39.1]	0.20 [0.12, 0.27]	0.345
MS (*SL_*B*_*)*	**0.82 [0.79, 0.85]**	**0.99 [0.96, 1.01]**	**0.16 [− 0.24, 0.57]**	**0.19 [0.17, 0.21]**	**31.2 [26.7, 35.7]**	**0.47 [0.38, 0.55]**	**0.487**
PD	0.82 [0.79, 0.85]	0.97 [0.94, 0.99]	0.15 [− 0.27, 0.56]	0.19 [0.17, 0.20]	29.7 [25.9, 33.4]	0.55 [0.48, 0.62]	0.537
PFF	0.75 [0.73, 0.78]	0.89 [0.88, 0.91]	0.14 [− 0.28, 0.56]	0.18 [0.16, 0.20]	32.2 [27.8, 36.6]	0.31 [0.20, 0.40]	0.448
SL_C_							
HA	0.81 [0.79, 0.83]	0.91 [0.90, 0.92]	0.10 [− 0.31, 0.50]	0.17 [0.16, 0.18]	29.0 [26.2, 31.8]	0.42 [0.36, 0.48]	0.509
CHF	0.93 [0.90, 0.95]	0.98 [0.96, 0.99]	0.05 [− 0.47, 0.56]	0.21 [0.19, 0.22]	30.3 [26.9, 33.8]	0.46 [0.39, 0.52]	0.473
COPD	0.85 [0.83, 0.87]	0.95 [0.94, 0.96]	0.10 [− 0.32, 0.52]	0.18 [0.17, 0.19]	26.9 [24.2, 29.6]	0.26 [0.19, 0.34]	0.420
MS	0.82 [0.79, 0.85]	0.95 [0.94, 0.97]	0.13 [− 0.32, 0.58]	0.20 [0.18, 0.22]	32.6 [27.9, 37.3]	0.22 [0.11, 0.32]	0.387
PD	0.82 [0.79, 0.85]	0.91 [0.89, 0.93]	0.09 [− 0.35, 0.53]	0.18 [0.17, 0.20]	30.5 [26.7, 34.4]	0.45 [0.37, 0.53]	0.498
PFF	0.75 [0.73, 0.78]	0.85 [0.84, 0.86]	0.09 [− 0.36, 0.54]	0.21 [0.19, 0.22]	34.5 [30.7, 38.4]	0.11 [0.00, 0.22]	0.328
SL_D_							
HA	0.81 [0.79, 0.83]	0.88 [0.86, 0.90]	0.07 [− 0.62, 0.76]	0.28 [0.26, 0.30]	41.9 [38.2, 45.5]	0.14 [0.06, 0.21]	0.250
CHF	0.93 [0.90, 0.95]	0.90 [0.88, 0.93]	− 0.03 [− 0.84, 0.79]	0.33 [0.30, 0.35]	42.3 [38.1, 46.6]	0.10 [0.01, 0.19]	0.205
COPD	0.85 [0.83, 0.87]	0.93 [0.91, 0.96]	0.08 [− 0.63, 0.80]	0.29 [0.27, 0.31]	40.4 [36.9, 43.9]	0.03 [− 0.05, 0.11]	0.200
MS	0.82 [0.79, 0.85]	0.92 [0.89, 0.96]	0.10 [− 0.59, 0.78]	0.28 [0.25, 0.30]	41.3 [35.2, 47.4]	0.15 [0.04, 0.26]	0.250
PD	0.82 [0.79, 0.85]	0.85 [0.82, 0.88]	0.03 [− 0.73, 0.79]	0.30 [0.28, 0.33]	44.9 [39.3, 50.5]	0.10 [0.00, 0.20]	0.225
PFF	0.75 [0.73, 0.78]	0.85 [0.81, 0.88]	0.09 [− 0.67, 0.85]	0.30 [0.27, 0.33]	47.7 [42.0, 53.5]	− 0.04 [− 0.14, 0.07]	0.172

Stride length obtained from the INDIP and the single wearable device, bias, limits of agreement (LoA) and intra class correlation (ICC_(2,1)_) for comparison between systems, and overall performance index for the SL algorithms. In italicface: recommended algorithms

*HA* healthy adults; *PD* Parkinson’s disease; *MS* multiple sclerosis; *COPD* chronic obstructive pulmonary disease; *CHF* congestive heart failure; *PFF* proximal femoral fracture; *CI* confidence intervals, *LoA* limits of agreement, *ICC* intra class correlation

The mean relative errors ranged from 25.3 to 34.1% for SL_A_, and similarly from 27.4 to 35.8% for SL_B_. These were larger for SL_C_ (ranging from 29.0 to 34.5%) and for SL_D_ (40.4 to 47.7%). The ICC_(2,1)_ for SL_A_ were the largest, ranging from 0.28 to 0.70, followed by SL_B_ with a range from 0.20 to 0.66. The ICC_(2,1)_ for SL_C_ were below 0.5, and below 0.15 for SL_D_.

Overall, **SL**_**A**_ presented the highest performance indexes for all cohorts excluding multiple sclerosis, with the following values: healthy older adults (0.582), congestive heart failure (0.663), chronic obstructive pulmonary disease (0.381), Parkinson’s disease (0.607), and proximal femoral fracture (0.465). In the multiple sclerosis cohort, **SL**_**B**_ had the highest performance index (0.487).

#### Effect of walking speed and bout duration

Critical errors in the stride length estimate were observed for the slowest bouts, with values decreasing below 20% only for walking speed > 0.5 m/s and below 10% only for 0.6 m/s (Fig. 3c). Highest errors were also still associated with shortest and slowest bouts (Fig. 4c); specifically, the shortest bouts (≤ 10 s) had a mean error of 32.6%, while the longest ones (> 60 s) 9.3%. Overall, errors higher than 50% were observed in about 17% of the total number of walking bouts. These bouts were short (13.03 ± 10.53 s), with slow walking speed (0.36 ± 0.13 m/s) and short stride length values (0.45 ± 0.17 m).

## Discussion

This is the first study presenting a comprehensive comparative assessment of a broad range of algorithms applied to a single wearable device, for estimating key digital mobility outcomes pertaining to gait (i.e., gait sequences, individual steps, cadence and stride length) in heterogeneous diseases and using data from the real world. In this work, we have described algorithms’ performances, selected the best algorithm for each digital mobility outcome and cohort, analysed the influence of walking speed and walking bout duration on their performance, and provided recommendations for their selection and implementation for real-world gait analysis.

### Gait sequence detection

When comparing all gait sequence detection algorithms, concurrent validity was high, reflected by ICC_(2,1)_ values and performance measures above 0.7, matching previous work [16, 41, 59]. Accuracy, specificity, and positive predictive values were very high for all gait sequence detection algorithms. Our results were comparable to previous work on a different population (post-stroke survivors) which reported similar sensitivity (0.92) and positive predictive value (0.84) of gait sequence detection algorithms implemented on data obtained from bilateral wearable devices on the feet [16]. The excellent results for specificity are similar or even higher than those reported previously in the literature 0.96 in Parkinson’s disease [59] and 0.93 in stroke survivors [16]. This is encouraging, as gait analysis relies on high specificity, which corresponds to a correct identification of gait sequences (high number of true positive events) while avoiding the misidentification of gait sequences (low number of false positive events). Avoiding incorrect identification of gait-sequences (as also reflected by positive predictive values) is preferable, to avoid the extraction of digital mobility outcomes from activities which are not directly representative of gait, such as shuffling or transitions [59].

When considering differences between algorithms, GSD_A_ and GSD_B_ tended to overestimate the total walking time (total gait sequence duration). This could potentially relate to different signal characteristics between the compared systems (low-back signals recorded with the wearable device may be different from feet signals [55] recorded with the INDIP). Slow gait, curved paths and short walking bouts with insufficient steady-pace phases for the spectral analysis could have also influenced the results, as the characteristics of the signals are more variable and the periods are less uniform than in steady-pace gait undertaken at faster speeds along straight paths [41].

Based on our findings collectively, we recommend using **GSD**_**B**_ on cohorts with slower gait speeds and substantial gait impairments (e.g., proximal femoral fracture). This may be because this algorithm is based on the acceleration norm (overall accelerometry signal rather than a specific axis/direction (e.g., vertical), hence it is more robust to sensor misalignments that are common in unsupervised real-life settings. Moreover, the use of adaptive threshold, that are derived from the features of a subject’s data and applied to step duration for detection of steps belonging to gait sequences, allows increased robustness of the algorithm to irregular and unstable gait patterns. **GSD**_**A**_ algorithm may be more suitable for cohorts with a faster gait speed and regular gait pattern (e.g., healthy older adults). This algorithm is based on a convolutional transformation (based on a gait cycle) of a single axis signal [40], potentially justifying its suitability to conditions characterised by more stable and regular gait patterns.

### Initial contact detection

Overall, all algorithms investigated for initial contact detection presented excellent sensitivity and positive predictive values (all above 0.81) and relative errors below 21% in diverse cohorts of patients. These errors are in line with previous work, although slightly higher than those assessed in laboratory or controlled and supervised environments, ranging between 4 and 13% [28, 39, 55]. Positive predictive values resulted were larger than sensitivity (although sensitivity values were > 0.75). This could be due to a lower number of false positive events (wrongly identified initial contact events) with respect to true positive events; slightly lower sensitivity measures reflect a higher number of missed initial contact events. Similar to gait sequence detection, higher positive predictive values (higher numbers of correctly identified initial contacts) are preferable, as gait assessment based on incorrectly identified events could lead to invalid digital mobility outcome extraction and misleading clinical interpretation. Low relative errors (< 11%), found for ICD_A_ and ICD_C,_ for step duration across all cohorts based on similar approaches are very encouraging and concurs with previous work which reported errors between 4 and 13% from data collected in laboratory conditions [39, 60].

Accurate detection of steps is critical for estimation of a plethora of digital mobility outcomes like cadence, step symmetry, gait variability, etc., which might have relevant clinical value (e.g., for the differentiation of stages of neurodegenerative diseases [60]). In addition, step detection can be used to refine the identification of gait sequences [41], and thus, the definition of a walking bout, which highlights the importance of using a robust algorithm with high sensitivity and positive predictive value.

For all cohorts, we recommend the use of the **ICD**_**A**_ for the identification of initial contact events, given the lowest absolute and relative errors (both in mean and standard deviation of step duration and initial contact time event) and best performance indexes. **ICD**_**A**_ is an optimized implementation of the algorithm based on continuous wavelet transform and peak detection originally presented in [42], and is frequently used and reported in the literature for heel-strike or initial time contact event detection [39, 61]. This algorithm has been previously validated under different conditions, producing similar results in algorithm performance [44] even if tested under less challenging conditions (such as supervised lab/clinical settings). To increase robustness to the variety of impaired gait patterns, **ICD**_**A**_ applies additional detrending and filtering before the continuous wavelet transform, then it detects the step-related peaks as maxima between zero-crossings (instead of using a predefined threshold for peak amplitude).

### Cadence estimation

The excellent performances of cadence algorithms, reflected by low relative errors of < 12%, were in line with [17, 41, 45], or lower than previous results reported in the literature (13–14%) [16]. As based upon [53], moderate to excellent ICC_(2,1)_ (> 0.70) were found in all cohorts except proximal femoral fracture, for the CAD_B_ and CAD_C_ algorithms. These results confirm the robustness of cadence estimation in all cohorts. Proximal femoral fracture data showed the lowest ICC_(2,1)_ values but good performances for the other metrics. This may be partially explained by the high asymmetry and the slow speed that characterize the proximal femoral fracture cohort (all proximal femoral fracture patients walked at a speed of < 1.29 m/s) [62]. This and the use of walking aids may have impacted the wearable device signal quality (amplitude and shape) and hence challenged the processing techniques on which the algorithms are based (i.e., wavelet transformations for CAD_A_ and CAD_B_ [41, 42], and zero-crossings for CAD_C_ [45]).

The recommended algorithm for cadence estimation is dependent upon the mobility function of the cohort. Overall, **CAD**_**C**_ performances were excellent across all cohorts, especially for groups with higher gait speeds. **CAD**_**B**_ was more robust in the proximal femoral fracture cohort as reflected by the performance index. Therefore, we suggest the implementation of **CAD**_**B**_ in cohorts with compromised gait speed and symmetry (e.g., severe or advanced neurological diseases) for which a zero-crossing approach may not be so suitable.

It is worth mentioning that the methodology for initial contact events/step detection, used by initial contact detection and cadence algorithms, includes two main stages. The first is related to the processing of the wearable device acceleration signal in order to remove noise, artefacts and to enhance the step-related features (e.g., zero-phase low-pass filtering, detrending). Then, on the processed acceleration signal, the initial contacts/steps are detected using peak detection or zero-crossing approaches. The combination of the various techniques for these two stages allowed us to implement optimized versions of state-of-the art algorithms.

Although initial contact detection and cadence algorithms are based on similar approaches, our results are in line with previous findings showing that the use of a peak detection approach may be more suitable for identification of events (initial contact detection), whereas zero-crossing techniques result in more accurate identification of cyclic events and step segmentation, required for the cadence estimation. All in all, as observed by Panebianco et al. [61], this underlines that each principle is better tailored to each digital mobility outcome; i.e., a wavelet transformation with peak detection is better suited for initial contact detection, whereas the zero-crossing approach seems better suited for the cadence.

### Stride length estimation

The performances of the stride length metrics are lower with respect to the other metrics presented in this work (e.g. cadence, initial contact detection), as reflected by relatively high absolute and relative errors, and low ICC_(2,1)_. This could be due to the nature of the lower-back accelerometry signals recorded in real-world conditions, from which the stride length is calculated. Particularly, the estimation of the position of the centre of mass (by double integration of the acceleration) and the inverted pendulum models on which stride length algorithms are based, assumes straight walking trajectories. Moreover these methodological principles do not consider turns or non-straight walking trajectories (i.e. veering). All of these deviations from a purely symmetrical and straight walking pattern are frequently found in real-world recordings [36].

Among the four algorithms, our recommendation is to use **SL**_**A**_ in all cohorts, given the lowest absolute, relative error and highest ICC_(2,1)_, as summarized by the performance indexes. It must be noted that SL_B_ was the best performer for the multiple sclerosis cohort, which is based on the same algorithm principle as SL_A_, but using a different correction factor implemented to estimate stride length [48]. All in all, SL_A_ showed good performance and similar to SL_B_ also for multiple sclerosis.

In general stride length algorithms tended to overestimate stride length between 0.07 and 0.16 m, this could be due to the correction factors that are implemented in both SL_A_ and SL_B_ [17]. Overall, the results highlight the better suitability of biomechanically-based algorithms, rather than those based solely upon machine-learning approaches. This is in line with the results observed on a previous study which implemented the same algorithms, trained on the same pre-available datasets [17]. This could be due to the fact that the biomechanically-based algorithms are less dependent on the intensity and morphology of the acceleration signals, and are highly influenced by the gait speed and irregularity of the gait patterns [17], which highlights a potential limitation in the generalization of the machine-learning based models when applied to external datasets. Future and novel machine-learning/deep-learning based models based on bigger datasets might produce better results.

The protocol used in the present study covered a comprehensive range of real-world scenarios. As such, results showed higher errors than those reported in previous studies: almost double with respect to [28] where results were evaluated from sensors on the shanks, and similar to [17] showing root mean square error between 0.04 and 0.18 m, where data was collected in the laboratory. This could potentially be due to the additional challenges involved in real-world and uncontrolled gait assessments presented in the current study, and the use of different data, i.e., based on a wearable device and on a different reference system for comparison. Moreover, to ensure a fair comparison of the algorithms, the walking bout (input) on which the algorithms were applied was defined and “imposed” by the reference system (INDIP). This could have potentially led to higher errors stemming from applying the algorithms to a wearable device signal with reduced amplitude and noisier characteristics with respect to the signal identified by the INDIP (sensors on the feet), especially for short and slow walking bout. All in all, our results highlight that future studies should focus on the development and optimization of stride length algorithms for increasing robustness of stride length estimation in order for this to be a useful (i.e., sensitive to change) digital mobility outcome that could be used in clinical interventional studies.

### Effect of walking speed and walking bout duration on algorithms’ performances

Generally, the performances of all algorithms significantly worsened for walking speeds below 0.5 m/s, which is considered as a threshold between slow and medium speed walkers [2], confirming what is well established in the literature [17, 63, 64]. This may be explained by the fact that the signals recorded with the wearable device in slow walkers are characterized by a compromised amplitude, non-uniform gait cycles [64, 65], and variable and irregular gait patterns [17]. Likewise, the lowest performances observed within proximal femoral fracture, may be explained by the lower speed and irregular gait patterns of this cohort [62]. Accordingly, the choice of algorithms for digital mobility outcome extraction should consider its sensitivity to gait speed, given its proven confounding effect on gait analysis [66], and the population of interest.

Walking bout duration also significantly affected the performances of the cadence algorithms, with an overall significant reduction of the relative error observed for longer walking bouts when estimating both step duration and cadence. This trend was also likely magnified by the fact that the shortest bouts were also the slowest ones and confirms similar previous results [34]. This could also be due to the fact that the impact of breaks (start and stop) and/or mis-detected strides in short walking bouts may be much larger than in longer walking bouts when quantifying algorithms’ performances.

Individual relative errors for stride length were higher for short walking bouts (e.g., < 10 s), although the median error did not seem to be significantly affected by bout length. digital mobility outcomes estimated from short walking bouts, which have been reported as the majority (about 50%) in real-world conditions [21, 67], should have special consideration as, in agreement with previous work, these walking bouts were observed to be the slowest [67], and therefore more sensitive to higher error estimation.

### General discussion

When considering the optimal location of the sensor, the signals recorded at the lower back are less robust than at other locations, such as the foot or shank, for the identification of initial contact events [61], although still more accurate than wrist data [68]. However, the lower back is among the most clinically favourable location for a single device, given its cost (one device), its location near to the centre of mass (which represents the overall human motion pattern), ergonomic conditions when worn attached to a belt or affixed to the skin, and its clinical value for fall risk, trunk stability and balance control, among others [21, 60, 69].

An advantage of real-world gait monitoring is the possibility of capturing a large number of diverse walking bouts and truly unsupervised gait performance in an ecologically valid environment [20]. However, the presence of contextual factors in a real-world context, which were not accounted for in this study, may have significantly influenced the performance of the algorithms. In particular, the presence of turns, the deviation from a straight path or other gait tasks (e.g., slope, presence of stairs or/and obstacles, crowdedness of space, visibility of trajectory), and the usage of various walking aids may have altered the gait pattern of the participant [20] and may partially explain the larger errors observed for stride length.

When comparing the performance between spatial and temporal digital mobility outcomes, the results indicate that the temporal characteristics (initial contact events, step duration, cadence) of gait, analysed with the proposed algorithms were more robust and valid than the spatial ones. This may be due to the fact that lower-back signals are better tailored to estimate particular events in the signal (i.e., initial contact events) and to assess its periodicity (i.e., cadence estimation) than to estimate displacements. These aspects should be considered when using the proposed algorithms, especially when interpreting findings for clinical applications and assessing minimal detectable changes in pathological gait. Moreover, it should be noted that given the biomechanical relationship between temporal and spatial features of gait, the identification of temporal estimates may directly impact on spatial calculations [48].

### Limitations

The results presented here are derived from real-world data comparing outcomes from a single wearable device to a reference system, INDIP, that has been thoroughly characterized and validated in a laboratory context, against a stereophotogrammetric system [23]. We did not include validation of DMOs derived from the single wearable device against a laboratory-based reference system as the focus of this study was on real-world gait. It must be noted that a complete algorithm ranking methodology should not only consider the overall findings for each cohort (as in this study) but should also consider the performance of algorithms on stratified subgroups (e.g., based on gait speed: slow-medium-fast walkers). This can be done by assigning a higher weight to the slow walkers’ results, given that their corresponding signals are more challenging and yield higher errors, as observed in this study. In addition, the percentage of walking bouts, as well as participants, in which the algorithm successfully provided digital mobility outcomes estimates should be considered to scale the overall performance of algorithms [24]. Thus, a simplified, although comprehensive, implementation of the ranking methodology could be seen as a limitation of this study. Nonetheless, the purpose of this was to provide an overall recommendation on the algorithm that performed best for each digital mobility outcome assessed in challenging real-world environments [20]. We are aware that, using a 2.5-h window of activity in the real world for the validation purposes, we may not have captured change and higher variability in mobility that are due to fatigue or the cyclic nature of activity. We also suggest that the inclusion of laboratory assessments for the implementation of the ranking methodology could be relevant. Indeed, even if collected under controlled or semi-structured conditions, data from short and slow walking bouts, that are typical in lab-based settings, may add variability and challenge algorithm performance [19]. In addition, the effect of walking aid use on results has not been assessed in this study. Thus, future work assessing this aspect could be clinically relevant, given the potential impact that walking aids (and the variety of types of walking aids) have on the quality of the wearable device signals and reference data [17], and as a consequence, on the assessment of the algorithm’s performance.

## Conclusions

This work was aimed at providing recommendations to implement and select algorithms for real-world gait analysis using lower-back worn sensors in patient cohorts with mobility impairments. We achieved this by comprehensively assessing and ranking algorithms’ performances, and we evaluated the effect of walking speed and walking bout duration on those performances.

The results highlighted good to excellent performances of the top algorithms in all cohorts. Particularly, algorithms for cadence and initial contact event detection were the most robust for all cohorts. Performances on gait sequence detection showed good performance measures, particularly when assessing sensitivity (> 0.70), positive predictive value (> 0.80), accuracy (> 0.95) and specificity (> 0.97). However, stride length estimation was the most challenging digital mobility outcome to estimate (with absolute error < 0.21 m). Relative errors for step duration and cadence generally decreased for longer walking bouts. Lower gait speeds (below 0.5 m/s) negatively influenced step duration, cadence and stride length estimates. We identified two top-performer algorithms for gait sequence detection [16] and cadence [45, 46], and a single best performer for initial contact detection [16] and stride length [47, 48]. The proximal femoral fracture cohort was the most challenging for algorithm performance.

In conclusion, the identified algorithms allow a robust estimation of digital mobility outcomes and gait characterization, with potential for improvement identified for stride length. Throughout this study we made recommendations for algorithm selection and implementation. Thus, our findings can be used to support future choices of the most suitable algorithms for real-world gait analysis, depending on type of cohort and research question. Finally, these results may inform future design of novel and more efficient gait analysis algorithms.

### Supplementary Information

**Additional file 1.** Details of ranking methodology.

## Data Availability

Representative data from the dataset and recommended algorithms presented in this study can be found on online repositories: Data can be found on Zenodo, 10.5281/zenodo.7547125. Algorithms can be found on GitHub, 10.5281/zenodo.7575872.

## Abbreviations

CAD: Cadence estimation
CI: Confidence intervals
CHF: Congestive heart failure
COPD: Chronic obstructive pulmonary disease
*d*: Duration
FP: False positive
FN: False negative
GSD: Gait sequence detection
HA: Healthy adults
ICC_(2,1)_: Intra-class correlation coefficient
ICD: Initial contact detection
INDIP: INertial modules, DIstance Sensors and Pressure insoles
LoA: Limits of agreement
MS: Multiple sclerosis
PD: Parkinson’s disease
PFF: Proximal femoral fracture
SL: Stride length estimation
TN: True negative
TP: True positive
WB: Walking bout
WBD: Walking bout duration
WD: Wearable device
*ws*: Walking speed
FIR: Finite impulse response filter
