# Correction: Assessing real-world gait with digital technology? Validation, insights and recommendations from the Mobilise-D consortium

**DOI:** 10.1186/s12984-024-01361-6

**Published:** 2024-05-03

**Authors:** M. Encarna Micó-Amigo, Tecla Bonci, Anisoara Paraschiv-Ionescu, Martin Ullrich, Cameron Kirk, Abolfazl Soltani, Arne Küderle, Eran Gazit, Francesca Salis, Lisa Alcock, Kamiar Aminian, Clemens Becker, Stefano Bertuletti, Philip Brown, Ellen Buckley, Alma Cantu, Anne-Elie Carsin, Marco Caruso, Brian Caulfield, Andrea Cereatti, Lorenzo Chiari, Ilaria D’Ascanio, Bjoern Eskofier, Sara Fernstad, Marcel Froehlich, Judith Garcia-Aymerich, Clint Hansen, Jeffrey M. Hausdorff, Hugo Hiden, Emily Hume, Alison Keogh, Felix Kluge, Sarah Koch, Walter Maetzler, Dimitrios Megaritis, Arne Mueller, Martijn Niessen, Luca Palmerini, Lars Schwickert, Kirsty Scott, Basil Sharrack, Henrik Sillén, David Singleton, Beatrix Vereijken, Ioannis Vogiatzis, Alison J. Yarnall, Lynn Rochester, Claudia Mazzà, Silvia Del Din, for the Mobilise-D consortium

**Affiliations:** 1 Translational and Clinical Research Institute, Faculty of Medical Sciences, Newcastle University, Newcastle Upon Tyne, UK; 2 Department of Mechanical Engineering and Insigneo Institute for in Silico Medicine, The University of Sheffield, Sheffield, UK; 3 Laboratory of Movement Analysis and Measurement, Ecole Polytechnique Federale de Lausanne, Lausanne, Switzerland; 4 Machine Learning and Data Analytics Lab, Department of Artificial Intelligence in Biomedical Engineering, Friedrich-Alexander-Universität Erlangen Nürnberg, Erlangen, Germany; 5 Center for the Study of Movement, Cognition and Mobility, Neurological Institute, Tel Aviv Sourasky Medical Center, Tel Aviv, Israel; 6 Department of Biomedical Sciences, University of Sassari, Sassari, Italy; 7 National Institute for Health and Care Research (NIHR) Newcastle Biomedical Research Centre (BRC), Newcastle University and The Newcastle Upon Tyne Hospitals NHS Foundation Trust, Newcastle Upon Tyne, UK; 8 Robert Bosch Gesellschaft für Medizinische Forschung, Stuttgart, Germany; 9 Department of Electronics and Telecommunications, Politecnico di Torino, Turin, Italy; 10 The Newcastle Upon Tyne Hospitals NHS Foundation Trust, Newcastle Upon Tyne, UK; 11 School of Computing, Newcastle University, Newcastle Upon Tyne, UK; 12 Barcelona Institute for Global Health (ISGlobal), Barcelona, Spain; 13 Universitat Pompeu Fabra, Barcelona, Catalonia Spain; 14 CIBER Epidemiología y Salud Pública (CIBERESP), Madrid, Spain; 15 Insight Centre for Data Analytics, University College Dublin, Dublin, Ireland; 16 School of Public Health, Physiotherapy and Sports Science, University College Dublin, Dublin, Ireland; 17 Department of Electrical, Electronic and Information Engineering «Guglielmo Marconi», University of Bologna, Bologna, Italy; 18 Health Sciences and Technologies-Interdepartmental Center for Industrial Research (CIRI-SDV), University of Bologna, Bologna, Italy; 19 Grünenthal GmbH, Aachen, Germany; 20 Department of Neurology, University Medical Center Schleswig-Holstein Campus Kiel, Kiel, Germany; 21 Sagol School of Neuroscience and Department of Physical Therapy, Sackler Faculty of Medicine, Tel Aviv University, Tel Aviv, Israel; 22 Rush Alzheimer’s Disease Center and Department of Orthopaedic Surgery, Rush University Medical Center, Chicago, IL USA; 23 Department of Sport, Exercise and Rehabilitation, Northumbria University Newcastle, Newcastle Upon Tyne, UK; 24 Novartis Institutes of Biomedical Research, Novartis Pharma AG, Basel, Switzerland; 25 McRoberts BV, The Hague, Netherlands; 26 Department of Neuroscience and Sheffield, NIHR Translational Neuroscience BRC, Sheffield Teaching Hospitals NHS Foundation Trust, Sheffield, UK; 27 Digital Health R&D, AstraZeneca, Stockholm, Sweden; 28 Department of Neuromedicine and Movement Science, Norwegian University of Science and Technology, Trondheim, Norway

**Correction: Journal of NeuroEngineering and Rehabilitation (2023) 20:78** 10.1186/s12984-023-01198-5

Following publication of the original article [1], the author noticed the errors in Table 1, and in Discussion section.

In Table 1 under Metric (Gait sequence detection) column, the algorithms GSD_B_ was updated with wrong description, input, output, language and citation and GSD_c_ with wrong description has been corrected as shown below:

**Table 1:** Description of algorithms for each metric: gait sequence detection (GSD), initial contact event detection (ICD), cadence estimation (CAD) and stride length estimation (SL)

Metric	Name	Description	Input	Output	Language	References
***Gait Sequence Detection***	GSD_A_	Based on a frequency-based approach, this algorithm is implemented on the vertical and anterior–posterior acceleration signals. First, these are band pass filtered to keep frequencies between 0.5 and 3 Hz. Next, a convolution of a 2 Hz sinewave (representing a template for a gait cycle) is performed, from which local maxima will be detected to define the regions of gait	***acc_v*:** vertical acceleration ***acc_ap*:** anterior–posterior acceleration ***WinS* = *3 s;*** window size for convolution ***OL* = *1.5 s; ***overlap of windows ***Activity_thresh* = *0.01;*** Motion threshold ***Fs:*** sampling frequency	***Start: ***beginning of N gait sequences [s] relative to the start of a recording or a test/trial. Format: 1 × N vector ***End: ***termination of N gait sequences [s] relative to the start of a recording or a test/trial. Format: 1 × N vector	Matlab®	Iluz, Gazit [40]
	GSD_B_	This algorithm, based on a time domain-approach, detects the gait periods based on identified steps. First, the norm of triaxial acceleration signal is low-pass filtered (*FIR*, *fc* = 3.2 Hz), then a peak detection procedure using a threshold of 0.1 [g] is applied to identify steps. Consecutive steps, detected using an adaptive step duration threshold are associated to gait sequences	***acc_norm*:** norm of the 3D-accelerometer signal ***Fs:*** sampling frequency ***th: ***peak detection threshold: 0.1 (g)	***Start: ***beginning of N gait sequences [s] relative to the start of a recording or a test/trial. Format: 1 × N vector ***End: ***termination of N gait sequences [s] relative to the start of a recording or a test/trial. Format: 1 × N vector	Matlab®	Paraschiv-Ionescu, Newman [41]
	GSDc	This algorithm utilizes the same approach as GSD_B _the only difference being a different threshold for peak detection of 0.15 [g]	***acc_norm*:** norm of the 3D-accelerometer signal ***Fs:*** sampling frequency ***th: ***peak detection threshold: 0.15 (g)	***Start: ***beginning of N gait sequences [s] relative to the start of a recording or a test/trial. Format: 1 × N vector ***End: ***termination of N gait sequences [s] relative to the start of a recording or a test/trial. Format: 1 × N vector	Matlab®	Paraschiv-Ionescu, Newman [41]

In Discussion section, the paragraph should read as "Based on our findings collectively, we recommend using **GSD**_**B**_ on cohorts with slower gait speeds and substantial gait impairments (e.g., proximal femoral fracture). This may be because this algorithm is based on the acceleration norm (overall accelerometry signal rather than a specific axis/direction (e.g., vertical), hence it is more robust to sensor misalignments that are common in unsupervised real-life settings. Moreover, the use of adaptive threshold, that are derived from the features of a subject’s data and applied to step duration for detection of steps belonging to gait sequences, allows increased robustness of the algorithm to irregular and unstable gait patterns" instead of “Based on our findings collectively, we recommend using **GSD**_**B**_ on cohorts with slower gait speeds and substantial gait impairments (e.g., proximal femoral fracture). This may be because this algorithm is based on the acceleration norm (overall accelerometry signal rather than a specific axis/direction (e.g., vertical), hence it is more robust to sensor misalignments that are common in unsupervised real-life settings [41]. Moreover, the use of adaptive thresholds, that are derived from the features of a subject’s data and applied to the amplitude of acceleration norm and to step duration for detection of steps belonging to gait sequences, allows increased robustness of the algorithm to irregular and unstable gait patterns”.
