# Supplementary material for: Assessing real-world gait with digital technology? Validation, insights and recommendations from the Mobilise-D consortium
Source: J Neuroeng Rehabil. 2023 Jun 14;20:78. doi: 10.1186/s12984-023-01198-5 (PMC10265910; doi:10.1186/s12984-023-01198-5)
Supplement: Supplementary file 1 — Additional file 1. Details of ranking methodology. [file 12984_2023_1198_MOESM1_ESM.docx]

# Additional File

In this file we indicate the nature of the performance measures (i.e., validity and performance measures) as a cost (c) or benefit (b) of the analyses included in the ranking methodology of the algorithms for each metric. Considering that each analysis has a relative contribution to the total performance index of a specific metric, here we also report the weights assigned to each analysis for each metric. Note that the weights assigned to the analyses are based on the consensus obtained within the Mobilise-D consortium (see Bonci et al. for more details on the calculation of each weight). Abbreviations: ICC: Intra Class Correlation, GSD: Gait Sequence Duration, SD: Standard Deviation, ICD: Initial Contact Detection, CAD: Cadence estimation, SL: Stride Length estimation.

**GSD performance measures**:

|  | **Analyses** | **Weights** |
| --- | --- | --- |
| **GSD** | Sensitivity (b) | 0.117 |
|  | Positive Predictive Value (b) | 0.105 |
|  | Accuracy (b) | 0.160 |
|  | Specificity (b) | 0.178 |
| **GSD - TOTAL** | Absolute Error [s] (c) | 0.081 |
|  | ICC (2,1) - Per participant (b) | 0.065 |
| **GSD - MEAN** | Absolute Error [s] (c) | 0.081 |
|  | ICC (2,1) - Per participant (b) | 0.065 |
| **GSD - SD** | Absolute Error [s] (c) | 0.081 |
|  | ICC (2,1) - Per participant (b) | 0.065 |

**ICD performance measures:**

|  | **Analyses** | **Weights** |
| --- | --- | --- |
| **ICD** | Sensitivity (b) | 0.237 |
|  | Positive Predictive Value (b) | 0.229 |
| **TIME EVENT (based on TP)** | Absolute Error [s] (c) | 0.267 |
|  | Relative Error [%] (c) | 0.267 |

TP: True Positive Events

**CAD performance measures:**

|  | **Analyses** | **Weights** |
| --- | --- | --- |
| **CADENCE** | Absolute Error [steps/min] (c) | 0.277 |
|  | Relative Error [%] (c) | 0.277 |
|  | ICC (2,1) - Per WB (b) | 0.446 |

**SL performance measures:**

|  | **Analyses** | **Weights** |
| --- | --- | --- |
| **STRIDE LENGTH** | Absolute Error [m] (c) | 0.277 |
|  | Relative Error [%] (c) | 0.277 |
|  | ICC (2,1) - Per WB (b) | 0.446 |

**Additional Table.** **Gait Sequence Detection (GSD) performance measures with additional analyses.** Gait sequence duration (mean and standard deviation) obtained from the INDIP and the single wearable device, bias, limits of agreement (LoA) and Intra Class Correlation (ICC _(2,1)_) for comparison between systems, and overall performance index for the GSD algorithms. Values are expressed as mean and 95% confidence intervals (CI) for each cohort. In boldface and italics recommended algorithms. Underlined Performance index indicates top ranked algorithm.

| **Cohort** | **MEAN GAIT SEQUENCE DURATION** | | | | | **SD of GAIT SEQUENCE DURATION** | | | | | **PERFORMANCE INDEX** |
| --- | --- | --- | --- | --- | --- | --- | --- | --- | --- | --- | --- |
|  | **INDIP  Mean and CI**  **[s]** | **Single wearable device  Mean and CI**  **[s]** | **Bias and LoA**  **[s]** | **Absolute Error [s]** | **ICC (2,1) (per participant)** | **INDIP  Mean and CI**  **[s]** | **Single wearable device  Mean and CI**  **[s]** | **Bias and LoA**  **[s]** | **Absolute Error [s]** | **ICC (2,1)** | **Combination of Measures** |
| **GSD_A_** | | | | | | | | | | |  |
| **HA** | **30.7 [18.9, 42.4]** | **26.5 [18.0, 35.0]** | **-4.1 [-18.9, 10.6]** | **4.7 [1.4, 8.1]** | **0.93 [0.83, 0.97]** | **68.0 [35.4, 100.5]** | **58.6 [32.1, 85.1]** | **-9.3 [-39.9, 21.3]** | **9.7 [2.5, 16.9]** | **0.96 [0.90, 0.98]** | **0.819** |
| **CHF** | **30.2 [16.6, 43.7]** | **28.2 [15.6, 40.7]** | **-2.0 [-9.8, 5.8]** | **3.4 [1.6, 5.3]** | **0.97 [0.90, 0.99]** | **52.8 [16.4, 89.2]** | **49.5 [11.9, 87.1]** | **-3.3 [-12.2, 5.6]** | **4.2 [1.7, 6.8]** | **0.99 [0.98, 1.00]** | **0.853** |
| **COPD** | **17.6 [15.0, 20.2]** | **16.6 [13.1, 20.1]** | **-1.0 [-8.1, 6.0]** | **2.5 [1.1, 3.9]** | **0.82 [0.57, 0.93]** | **26.1 [14.5, 37.8]** | **25.7 [12.3, 39.1]** | **-0.5 [-26.5, 25.6]** | **6.5 [0.6, 12.4]** | **0.86 [0.66, 0.95]** | **0.822** |
| **MS** | **25.7 [20.3, 31.1]** | **25.6 [21.1, 30.1]** | **-0.2 [-16.5, 16.2]** | **4.4 [1.0, 7.9]** | **0.67 [0.32, 0.86]** | **48.4 [31.0, 65.7]** | **51.9 [35.3, 68.6]** | **3.6 [-50.3, 57.4]** | **12.3 [0.0, 24.6]** | **0.69 [0.35, 0.87]** | **0.735** |
| **PD** | **32.6 [22.0, 43.2]** | **30.6 [21.7, 39.5]** | **-2.0 [-16.0, 12.0]** | **4.7 [2.0, 7.4]** | **0.94 [0.85, 0.98]** | **65.4 [25.9, 104.9]** | **56.6 [22.3, 90.9]** | **-8.8 [-37.4, 19.8]** | **9.6 [2.8, 16.4]** | **0.98 [0.94, 0.99]** | **0.852** |
| PF**F** | 26.8 [19.4, 34.1] | 18.9 [14.0, 23.8] | -7.9 [-26.2, 10.4] | 8.0 [2.9, 13.1] | 0.49 [0.01, 0.79] | 32.2 [20.9, 43.4] | 24.3 [13.9, 34.7] | -7.8 [-21.7, 6.0] | 7.9 [4.1, 11.8] | 0.86 [0.65, 0.95] | 0.770 |
| **GSD_B_** | | | | | | | | | | |  |
| HA | 31.4 [19.1, 43.8] | 19.1 [13.3, 24.9] | -12.3 [-40.2, 15.6] | 12.3 [5.4, 19.2] | 0.61 [0.23, 0.83] | 70.7 [36.8, 104.6] | 48.7 [24.2, 73.2] | -22.0 [-64.1, 20.1] | 22.0 [11.7, 32.4] | 0.88 [0.72, 0.95] | 0.727 |
| CHF | 30.2 [16.6, 43.7] | 21.0 [10.5, 31.5] | -9.1 [-23.4, 5.2] | 9.2 [4.0, 14.3] | 0.79 [0.38, 0.94] | 52.8 [16.4, 89.2] | 38.5 [7.4, 69.7] | -14.3 [-35.3, 6.8] | 14.3 [6.6, 22.0] | 0.93 [0.77, 0.98] | 0.792 |
| COPD | 17.6 [15.0, 20.2] | 12.0 [9.7, 14.3] | -5.6 [-8.8, -2.5] | 5.6 [4.8, 6.5] | 0.44 [-0.03, 0.75] | 26.1 [14.5, 37.8] | 17.4 [10.0, 24.8] | -8.7 [-27.6, 10.2] | 8.7 [3.7, 13.6] | 0.78 [0.51, 0.92] | 0.814 |
| *MS* | *25.7 [20.3, 31.1]* | *18.4 [14.7, 22.0]* | *-7.4 [-25.1, 10.4]* | *9.9 [6.9, 12.9]* | *0.34 [-0.13, 0.68]* | *48.4 [31.0, 65.7]* | *41.5 [27.5, 55.6]* | *-6.8 [-63.6, 50.0]* | *19.5 [8.6, 30.5]* | *0.58 [0.18, 0.82]* | *0.655* |
| *PD* | *32.6 [22.0, 43.2]* | *23.7 [16.9, 30.4]* | *-8.9 [-28.9, 11.1]* | *10.2 [5.9, 14.5]* | *0.75 [0.47, 0.90]* | *65.4 [25.9, 104.9]* | *40.6 [22.5, 58.7]* | *-24.8 [-121.8, 72.1]* | *25.6 [1.9, 49.2]* | *0.65 [0.30, 0.85]* | *0.726* |
| **PFF** | **25.9 [18.9, 33.0]** | **16.2 [11.8, 20.7]** | **-9.7 [-24.9, 5.5]** | **9.9 [6.0, 13.9]** | **0.48 [0.01, 0.78]** | **30.9 [20.1, 41.7]** | **23.5 [14.7, 32.4]** | **-7.4 [-20.3, 5.6]** | **8.7 [6.3, 11.1]** | **0.87 [0.67, 0.95]** | **0.771** |
| GSD_C_ | | | | | | | | | | |  |
| HA | 31.4 [19.1, 43.8] | 20.9 [14.4, 27.4] | -10.5 [-36.3, 15.3] | 10.5 [4.2, 16.9] | 0.69 [0.36, 0.87] | 70.7 [36.8, 104.6] | 53.3 [26.7, 79.8] | -17.4 [-54.0, 19.1] | 17.5 [8.5, 26.4] | 0.92 [0.81, 0.97] | 0.722 |
| CHF | 30.2 [16.6, 43.7] | 22.6 [11.3, 33.9] | -7.6 [-24.5, 9.3] | 9.5 [5.1, 13.9] | 0.80 [0.42, 0.95] | 52.8 [16.4, 89.2] | 39.4 [7.6, 71.3] | -13.4 [-33.8, 7.0] | 14.1 [7.4, 20.8] | 0.94 [0.79, 0.98] | 0.811 |
| COPD | 17.6 [15.0, 20.2] | 11.7 [9.0, 14.5] | -5.9 [-9.8, -2.0] | 5.9 [4.8, 6.9] | 0.46 [0.01, 0.76] | 26.1 [14.5, 37.8] | 16.4 [7.9, 25.0] | -9.7 [-28.1, 8.7] | 9.7 [4.9, 14.5] | 0.79 [0.52, 0.92] | 0.776 |
| MS | 25.7 [20.3, 31.1] | 19.0 [15.1, 23.0] | -6.7 [-24.3, 10.9] | 9.3 [6.3, 12.3] | 0.40 [-0.06, 0.72] | 48.4 [31.0, 65.7] | 42.3 [27.2, 57.4] | -6.1 [-61.4, 49.3] | 18.4 [7.5, 29.2] | 0.63 [0.26, 0.84] | 0.693 |
| PD | 32.6 [22.0, 43.2] | 25.8 [18.8, 32.8] | -6.8 [-28.7, 15.1] | 9.1 [4.6, 13.6] | 0.77 [0.50, 0.90] | 65.4 [25.9, 104.9] | 42.3 [23.2, 61.3] | -23.2 [-132.6, 86.3] | 24.2 [-2.5, 50.9] | 0.59 [0.20, 0.82] | 0.726 |
| PFF | 26.8 [19.4, 34.1] | 16.5 [12.3, 20.7] | -10.3 [-30.6, 10.1] | 10.9 [5.6, 16.2] | 0.27 [-0.24, 0.68] | 32.2 [20.9, 43.4] | 23.6 [13.8, 33.5] | -8.5 [-27.4, 10.4] | 10.6 [6.7, 14.5] | 0.79 [0.49, 0.92] | 0.687 |

HA: Healthy adults; PD: Parkinson’s disease; MS: Multiple Sclerosis; COPD: Chronic Obstructive Pulmonary Disease; CHF: Congestive Heart Failure; PFF: Proximal Femoral Fracture; SD: Standard deviation, CI: Confidence interval, LoA: Limits of Agreement, ICC: Intra Class Correlation.
